# CardioGraph: a platform to study variations associated with familiar cardiopathies

**DOI:** 10.1186/s12911-024-02700-2

**Published:** 2024-10-21

**Authors:** Alberto García S., Mireia Costa, Ana Perez, Oscar Pastor

**Affiliations:** PROS Research Center, VRAIN Research Institute, Universitat Politècnica de València, Camino de Vera, Valencia, Spain

**Keywords:** Familiar cardiopathies, Novel variations, Knowledge representation, Conceptual modeling

## Abstract

**Background:**

Familiar cardiopathies are genetic disorders that affect the heart. Cardiologists face a significant problem when treating patients suffering from these disorders: most DNA variations are novel (i.e., they have not been classified before). To facilitate the analysis of novel variations, we present CardioGraph, a platform specially designed to support the analysis of novel variations and help determine whether they are relevant for diagnosis. To do this, CardioGraph identifies and annotates the consequence of variations and provides contextual information regarding which heart structures, pathways, and biological processes are potentially affected by those variations.

**Methods:**

We conducted our work through three steps. First, we define a data model to support the representation of the heterogeneous information. Second, we instantiate this data model to integrate and represent all the genomics knowledge available for familiar cardiopathies. In this step, we consider genomic data sources and the scientific literature. Third, the design and implementation of the CardioGraph platform. A three-tier structure was used: the database, the backend, and the frontend.

**Results:**

Three main results were obtained: the data model, the knowledge base generated with the instantiation of the data model, and the platform itself. The platform code has been included as supplemental material in this manuscript. Besides, an instance is publicly available in the following link: https://genomics-hub.pros.dsic.upv.es:3090.

**Conclusion:**

CardioGraph is a platform that supports the analysis of novel variations. Future work will expand the body of knowledge about familiar cardiopathies and include new information about hotspots, functional studies, and previously reported variations.

## Background

### Introduction

Familiar cardiopathies, also known as *inherited cardiovascular diseases*, are genetic-based disorders that affect the heart. These disorders are characterized by a heterogeneous clinical evolution, a strong familiar component, and an increased risk of sudden cardiac death [1]. Depending on how the heart is affected, there are three types of familiar cardiopathies: i) *cardiomyopathies*, which affect muscle structure and contraction process; ii) *channelopathies*, which affect the electrical activity of the heart; and iii) *genetic aortic diseases*, which affect the aorta, the largest artery in the human body.

Due to the genetic heterogeneity associated with familiar cardiopathies, cardiologists face a critical problem when working with patients suffering from cardiopathies: Most of the DNA variations they work with are novel (i.e., newly discovered) [2]. This means that these variations have not been clinically classified as pathogenic, uncertain significance (VUS), or benign. This problem makes it difficult for cardiologists to determine whether the origin of a cardiopathy is genetic, and they cannot provide an early diagnosis to patients and their relatives.

In terms of time and effort, variation classification is an expensive process. As a result, classifying all of the novel variations from a patient can take days to weeks, depending on the number of variations to evaluate. This work presents CardioGraph, a platform designed to prioritize variations clinically associated with familiar cardiopathies, reducing the number of novel variations cardiologists must evaluate and study. CardioGraph uses a complex workflow to annotate the consequences of the variations, focusing on how severe the effect on the protein’s structure or function is likely to be. This information is supplemented with contextual information about the heart structure, pathways, and affected pathways. With all this data, CardioGraph can identify variations more likely to be relevant in the context of familiar cardiopathies. Thanks to these capabilities, using CardioGraph, cardiologists can focus on variations that are more likely to be relevant for familiar cardiopathies, reducing workload and helping make well-informed clinical decisions.

To facilitate data retrieval, integration, and analysis, we have designed and implemented CardioGraph following a conceptual model-based approach. This allowed us to take advantage of the benefits associated with managing genomics data using data models [3, 4]. Currently, CardioGraph consists of three modules. A brief description is presented below:

- Data Model: The data model used to retrieve, integrate, and analyze genomics data associated with familiar cardiopathies is described.
- Knowledge Graph: The information associated with genetic aortic disorders, channelopathies, and cardiomyopathies is displayed in the form of a directed graph. The graph follows the data model presented in the previous module.
- The Platform: A service that annotates Variant Call Format (VCF) files, enriching the context of their variations. Each variation is annotated with: i) its consequence according to SnpEff [5], ii) the resulting amino acid change and its effect, and iii) plenty of contextual information provided by our knowledge graph.

CardioGraph has been developed and validated with the help and support of cardiologists from the Health Research Institute Hospital La Fe in Valencia, Spain, and the Institute of Health and Biomedical Research in Alicante, Spain.

### Related work

Knowledge graphs have gained popularity in recent years due to their ability to structure information and make complex connections explicit. Currently, they are being used in medicine to structure complex disease information, drug repurposing, and the biomedical literature [6]. In the particular context of cardiology disorders, knowledge graphs are useful for improving treatment decisions and patient management [7]. However, to the best of our knowledge, no knowledge graphs have been used for variation prioritization in familiar cardiopathies. Instead, we have found nine tools (The Atlas of Cardiac Genetic Variation, CardioClasiffier, CardioGenBase, Marfan Syndrome (FBN1), TTN database, Hypertrophic CardioMyopath (HCM) risk predictor, ARVC database, PhenoDis, and CardioVAI) for the prioritization and classification of variations in the context of familiar cardiopathies. Table 1 provides the general function of each tool and the link. Below, we provide a more detailed description of the functionalities of each tool.

**Table 1:** CardioGraph-like tools analyzed

Tool	Type	Link
ACGV [38]	Database	https://www.cardiodb.org/acgv/
CardioClassifier [39]	Classifier	https://www.cardioclassifier.org
CardioGenBase [40]	Database	http://www.cardiogenbase.com/ (Problematic url)
Marfan Syndrome (FBN1) [41]	Database	http://www.umd.be/FBN1/
TTN database [42]	Database	https://www.cardiodb.org/titin/index.php
HCM risk predictor [43]	Predictor	https://doc2do.com/hcm/webHCM.html
ARVC database [44]	Database	https://arvc.molgeniscloud.org
PhenoDis [45]	Database	http://mips.helmholtz-muenchen.de/phenodis/
CardioVAI [46]	Classifier	http://cardiovai.engenome.com (Not active url)

#### The Atlas of Cardiac Genetic Variation

The Atlas of Cardiac Genetic Variation (ACGV) is a database of variations associated with Hypertrophic Cardiomyopathy (HCM), Dilated Cardiomyopathy (DCM), and Arrhythmogenic Right Ventricular Cardiomyopathy (ARVC). This database offers information about the frequency of a variation in a population according to the Exome Aggregation Consortium (ExAC) [8], the clinical role of variations according to the Oxford Molecular Genetics Laboratory and the Laboratory of Molecular Medicine, and variation-specific information such as position and alleles. There are three ways to explore the information provided by the ACGV database: i) by studying all the variations associated with HCM, DCM, or ARVC, ii) by studying all the variations located in a particular gene, or iii) by studying all the information available about a particular variation.

#### CardioClassifier

CardioClassifier is a tool that supports variation interpretation by semi-automating the American College of Medical Genetics and Genomics and the Association for Molecular Pathology (ACMG/AMP) guidelines The Standards and Guidelines for the Interpretation of Sequence variations (i.e., ACMG/AMP guidelines) [9]. These guidelines are proposed by the American College of Medical Genetics and the Association for Molecular Pathology to interpret DNA variations in the context of inherited disorders. Worldwide, it is the most followed standard for variation interpretation. in the context of inherited cardiac conditions. The tool takes as input either a single variation or a VCF file, and the users can select the specific set of criteria from the ACMG/AMP guidelines they want to evaluate. Currently, it supports variation interpretation in up to 40 genes and 11 cardiac disorders.

#### CardioGenBase

CardioGenBase is a database that provides gene-disorder associations in the context of major cardiovascular disorders (e.g., cerebrovascular disorders and coronary artery disorders). Its knowledge comes from curating, integrating, and analyzing specialized literature from PubMed and MEDLINE. Specifically, it provides the following information: gene’s chromosomal position, ontology data, gene and protein expression patterns, information about pathways, and DNA variations. However, the link to CardioGenBase is malfunctioning, and it is not accessible.

#### Marfan Syndrome (FBN1)

The Marfan Syndrome (FBN1) database provides information about published DNA and protein variations affecting the FBN1 gene. Its objective is to facilitate the establishment of structure-function and phenotype-genotype relationships. For each variation available, it provides information at the gene, protein, and clinical levels. The information representation follows the HUGO Mutation Database Initiative [10] and the HGVS nomenclature standards.

#### TTN database

The TTN database describes truncating variations (i.e., variations that shorten the protein) in the TTN gene and their association with the Dilated Cardiomyopathy disorder. Specifically, it provides information about the TTN gene transcripts, the exons contained in each transcript, functional data (regions, domains, symmetry) about such exons, the DNA variations detected in each exon, and the cohort studies where the variations were detected. The Cardiovascular Genetics and Genomics Group (National Institute for Health Research, Imperial College London, and the Clinical Sciences Centre) created the database in conjunction with the European Bioinformatics Institute and TTN locus-specific database.

#### HCM risk predictor

The HCM risk predictor is the first validated risk predictor for Hypertrophic Cardiomyopathy patients’ risk of sudden cardiac death (SCD). Its predictions are based on the assessment of specific clinical parameters following the recommendations of the 2014 ESC Guidelines on Diagnosis and Management of Hypertrophic Cardiomyopathy [11]. Additionally, the presence or absence of such parameters can also be used to determine whether an implantable cardioverter-defibrillator (ICD) implantation is necessary.

#### ARVC database

The ARVD database provides information about DNA variations in genes associated with Arrhythmogenic Right Ventricular Cardiomyopathy (ARVC). They obtain information about the type of variation and protein level information from curated literature, clinical studies, and unpublished data referring to ARVC or their associated genes. All this collected data was transformed into the Leiden Open Variation Database (LOVD) [12] format. This database is helpful for experimenters and clinicians that seek to determine if a variation has been reported or considered pathogenic before.

#### PhenoDis

PhenoDis is a manually curated database containing information about 327 rare cardiac conditions. For each condition, they provide clinical data (prevalence, inheritance, age of onset, and symptoms, genetic information), disease-causing variations reported in ClinVar [13], and additional information of interest about diagnosis, clinical description, and molecular genetics. All this information will help develop computational risk predictors, decision support systems, and phenotype-driven strategies that intend to identify relevant genes.

#### CardioVAI

CardioVAI is a web tool that supports variation interpretation in the context of cardiovascular disorders. For such purpose, they have automated the ACMG/AMP guidelines [9], and their adaptation to the MYH7 gene variations [14]. The automation is based on the information available in public databases such as ClinVar [13], MedGen, ExAC [8], and Disease Ontology. For each variation, they provide information about the cardiovascular disorders potentially associated and the interpretation results according to the five-rank system proposed by the ACMG/AMP guidelines. However, the link to CardioVAI is malfunctioning, and the tool cannot be used at this moment.

## Materials and methods

### Data model

Genomics is a very complex domain. The dispersion of genomics data in thousands of repositories has caused the data to be highly heterogeneous in its representation, making considerably complex data integration and knowledge extraction. For instance, the Nucleic Acids Research (NAR) online Molecular Biology Database Collection contains 1.764 database after its last update this year [15].

Technological advancements have enabled the acquisition of various data types, such as whole genomes, epigenetic modifications, transcriptomic regulations, and protein-protein interactions, which promise to improve the quality of data analysis and knowledge generation. However, in addition to the problems mentioned above, genomics faces unique challenges related to the semantics of their data. One of these challenges is mapping identifiers between different omics platforms. For example, how to map and integrate a set of transcript identifiers with a set of protein identifiers, and it is not uncommon to come across molecules that cannot be mapped between two types of datasets [15].

In this context, following a conceptual modeling-based approach would help represent this heterogeneous information, achieving the needed semantic interoperability and facilitating knowledge extraction.

Consequently, we decided to introduce the conceptual modeling perspective in CardioGraph and represent all the information the platform will manage according to a well-known conceptual schema of the human genome: The Conceptual Schema of the Human Genome (CSHG) [3]. The CSHG covers several dimensions of genomics knowledge with a very detailed perspective. However, only some of the information represented in these dimensions is relevant for CardioGraph.

To simplify the CSHG and select only the relevant pieces of information, we used the ISGE method [16] that, given a conceptual schema, allows us to obtain a simplified version of such schema tailored to the user’s needs (note that the name of the method is an acronym based on the names of its phases, namely, Identify, Select, and GEnerate). The first phase of ISGE is identifying the essential components of the use case, which are the basic building units required to construct the simplified schema. The requirements for this first phase of ISGE (I phase) are expressed as a set of competency questions. A competency question (CQ) is a natural language sentence that describes a requirement to be answered. The competency questions arose from our collaboration with domain experts based on how they work with data and generate knowledge.

Q1: How is a gene affected by a variation?
Q2: How is a protein affected by a variation?
Q3: How is a biological pathway affected by a variation?
Q4: How is a particular heart biological element affected by a variation?

The other two phases of ISGE involve selecting relevant parts of the original conceptual schema (S phase) and generating the new, simplified conceptual schema while resolving any inconsistencies (GE phase). After applying the ISGE method to the CSHG, we obtained the data model supporting CardioGraph.

### Knowledge graph

Once the data model was defined, we instantiated it to represent the genomics knowledge associated with cardiomyopathies, channelopathies, and genetic aortic disorders. Such knowledge was obtained using two kinds of resources: i) well-known genomic databases and ii) scientific literature.

First, the following genomic databases were consulted: NCBI Gene, Uniprot [17], Reactome [18], and KEGG [19]. NCBI Gene [20] is a database from the National Center for Biotechnology Information containing information about genes. The database’s web search function was used to look for information about a each gene. The query employed follows the structure *“Homo Sapiens [gene symbol]”*, where *Homo Sapiens* represents the species of interest (e.g., human), and the *gene symbol* symbol (e.g., TTN) represents the common symbol of the gene we want to learn about. This query was run manually each time information about a gene was needed.

Uniprot [17] is a comprehensive database of sequence and functional data about proteins. Following the same approach as in the NCBI Gene database, a query was performed in the database’s web search function to obtain information about a protein of interest. The queries performed follow the structure *“(taxonomy_id:9606) AND (gene:[gene symbol])”*, where *taxonomy_id:9606* specifies we are interested in the human species, and the *gene symbol* represents the symbol of the gene that codifies for the protein of interest. Again, the query was manually executed each time information about a protein was required.

Finally, both Reactome [18] and KEGG [19] are databases containing information about pathways and the proteins and entities participating in them. In reactome, we used the web search service to obtain all the pathways in which a certain protein participates. Specifically, each search was performed using the Uniprot protein identifier (e.g., *Q8WZ42* for the TTN gene), which can be obtained directly from the Uniprot database when obtaining the information about the protein of interest. In the KEGG case, we perform a search by *gene symbol*. Then, in the search results we identified the entry in the *KEGG GENES* section referring to the gene of interest. In each gene page, the pathways associated are specified.

The information that can be obtained from these databases was not enough to obtain all the knowledge that is behind familiar cardiopathies. Consequently, we curated specialized scientific literature to complete the information obtained from the databases mentioned above. Table 2 presents the literature consulted for cardiomyopathies, channelopathies, and genetic aortic diseases.

**Table 2:** The list of consulted literature for generating the knowledge bases

Title	Reference
**Cardiomyopathy Disorders**	
Cardiac Titin and Heart Disease	[47]
Cardiac myosin-binding protein C (MYBPC3) in cardiac pathophysiology	[48]
Sarcomeric proteins and inherited cardiomyopathies	[49]
Sarcomere Imaging by Quantum Dots for the Study of Cardiac Muscle Physiology	[50]
Sarcomeric Protein Isoform Transitions in Cardiac Muscle: A Journey to Heart Failure	[51]
Overview of the Muscle Cytoskeleton	[52]
The Sarcomeric Z-Disc and Z-Discopathies	[53]
The M-band: The underestimated part of the sarcomere	[54]
Cardiac Disorders and Pathophysiology of Sarcomeric Proteins	[55]
Contemporary Definitions and Classification of the Cardiomyopathies	[56]
Guyton and Hall Textbook of Medical Physiology	[24]
The sliding filament theory of muscle contraction	[57]
**Channelopathy Disorders**	
Cardiac Ion Channels	[31]
The Physiology and Pathophysiology of T-Tubules in the Heart	[58]
Ion Channels in the Heart	[59]
The Cardiac Conduction System	[60]
Channelopathies	[61]
Genetics and cardiac channelopathies	[62]
**Aortic Disorders**	
Micromechanics of elastic lamellae: unravelling the role of structural inhomogeneity in multi-scale arterial mechanics	[27]
Fascia: The Tensional Network of the Human Body	[29]
Fibrillin microfibrils and elastic fibre proteins: Functional interactions and extracellular regulation of growth factors	[30]
Extracellular matrix, regional heterogeneity of the aorta, and aortic aneurysm	[63]
The role of TGF-$\beta$ signaling in myocardial infarction and cardiac remodeling	[64]

### The platform

**Fig. 1:**
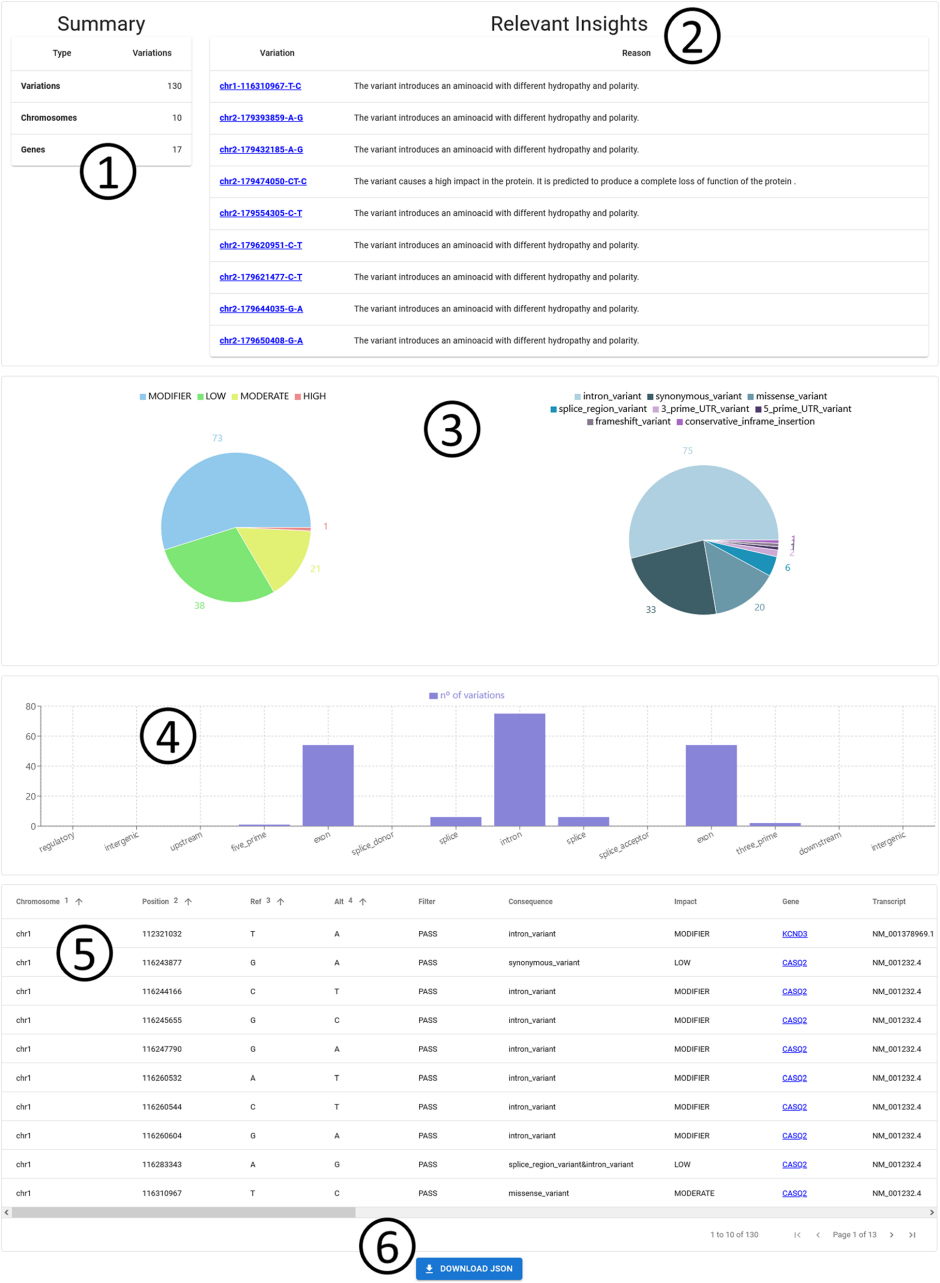
User interface displayed after analyzing the VCF file. 1: a summary containing the list of variations. 2: A list of variations considered to be relevant by CargioGraph. 3: two charts representing the consequences of the identified DNA variations. 4: the distribution of variations among the different regions of the genome. 5: a grid containing all the variations with their associated information. 6: a button to download the generated insights

The CardioGraph platform has been developed following a three-tier architecture. In web application development, a three-tier architecture is a typical design pattern. It divides the application into three logical layers, each with a distinct role and set of responsibilities. These layers are as follows: database layer, backend layer, and frontend layer. The database layer is responsible for data storage and retrieval. This layer has been constructed as a graph database using Neo4J. The backend layer contains the application’s core logic, which processes user requests from the frontend layer and performs the required tasks. Specifically, this module is responsible of processing and filtering VCFs, annotating VCFs using the SnpEff tool and selecting the most relevant variations following a set of pre-defined rules. We considered a variation to be relevant when: i) it is predicted to have a HIGH impact on the transcript according to SnpEff, ii) it causes a disruptive change in the protein sequence, iii) it causes LOF or NMD, or iv) it causes a disruptive change in the protein sequence, or it introduces a critical amino acid change, i.e., there is a change in both hydropathy and polarity compared to the original amino acid (see excerpt 2 in Fig. 1)

The SnpEff annotation tool was choosen ahead other well-known tools such as VEP [21] or Annovar [22] because is the only one that provides information about whether the variation causes a loss-of-function (LOF) or nonsense mediated decay (NMD) [21], which is one of the rules evaluated to identify the relevant variations. The backend layer has been developed in JavaScipt with the Node.js runtime environment. Finally, The frontend layer handles user interface interactions, displays information to users, and receives input from them. This layer has been developed in JavaScript with the React.js library.

## Results

### CardioGraph data model

**Fig. 2:**
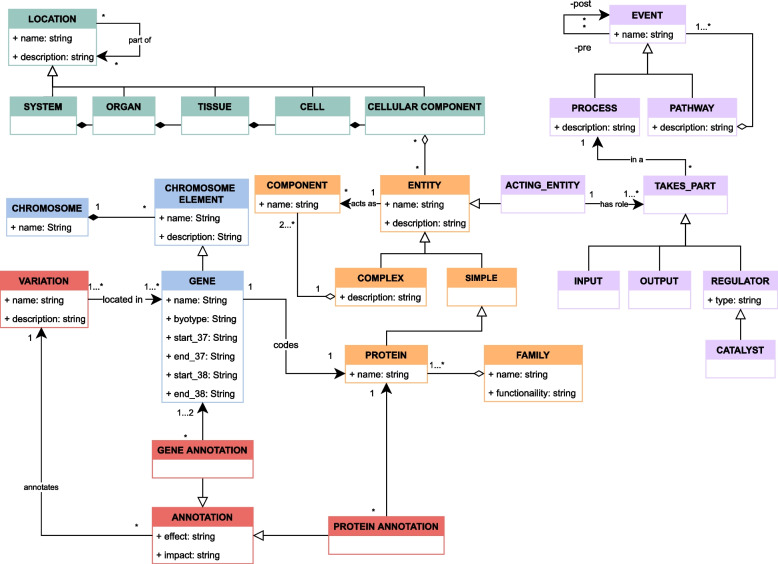
Conceptual schema for cardiopathies obtained using the ISGE method. Red: structural-related classes. Orange: entity-related classes. Green: Location-related classes. Lilac: pathway-related classes

The ISGE method was applied to the CSHG to obtain a simplified conceptual schema tailored to CardioGraph’s information needs, as described in Data model section. Figure 2 shows the resulting conceptual schema, which we called *the Conceptual Schema of Cardiopathies* (CSC).

The classes that compose the CSC are grouped into five dimensions:

1. The **Location dimension** describes body locations and will allow the representation of the heart’s morphology during the instantiation process. The Locations have been defined as a hierarchical composition that goes from the more general to the more specific. First, there are Systems, which cluster Organs that provide specific functionality. Every Organ is composed of different types of Tissues, which are made of millions of Cells. Cells, in turn, are constituted by biomolecules and structures called Cellular components.
2. The **Entity dimension** describes the biological entities of our body and will allow the representation of proteins, complexes, and other components relevant to the familiar cardiopathies’ understanding. A biological Entity is considered Complex when it can be decomposed into smaller pieces that act as Components. Otherwise, it is considered Simple. There are different types of Simple entities, but one of the most important is the Proteins because they play essential roles in our body’s function, regulation, and structure. Usually, Proteins are grouped into Families by functionality. Other examples of Simple entities include ions and molecules such as ATP or ADP (e.g., Ions trigger some processes of the heart’s muscle contraction while ATP and ADP are used by cells to obtain energy).
3. The **Structural dimension** describes the functional regions of DNA and will allow the representation of the genes that codify proteins with a structural or functional role in the heart. Our DNA is arranged in Chromosomes, that contain different Chromosome element with specific functionalities. One of these elements is the Gene, which is of high importance due to its function: codifying proteins.
4. The **Pathway dimension** describes the interactions of biological Entities and will allow the representation of the events and pathways that can be potentially affected by familiar cardiopathies. Compliant with the bio notation, these Events can be either a Pathway or a Process, depending on whether they can be decomposed in simpler Events. We defined non-rigid specializations [23] of the Entity, called Acting Entity. These Acting entities must Take part in at least one specific Process as either an Input, an Output, or a Regulator. This characterization allows us to differentiate between entities that *exist* and entities that *participate* in processes.
5. The **Variation dimension** describes the Variations that occur in the DNA and their consequence in Genes and Proteins by means of Annotations. Those Annotations that predict the impact of a Variation at the Gene level are called Gene annotation, and those that predict the impact at the protein level are called Protein annotation. This dimension will allow the representation of the DNA variations relevant for diagnosing or treating familiar cardiopathies.

### CardioGraph knowledge graph

**Fig. 3:**
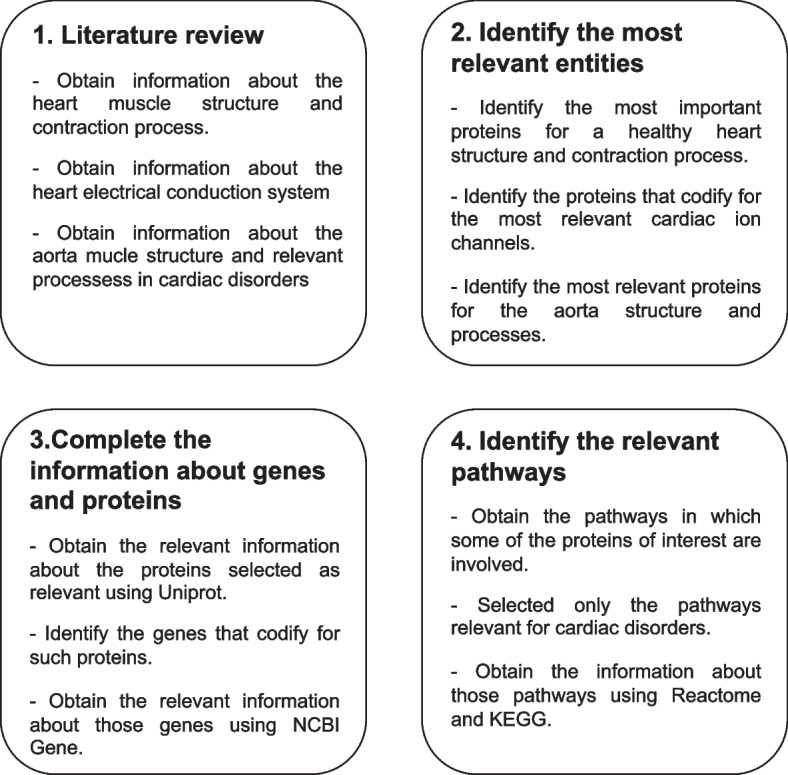
Processed followed to carry out the instantiation of cardiograph data model

The CardioGraph data model was instantiated to represent the genomics knowledge gathered about cardiomyopathies, channelopathies, and genetic aortic diseases. Such knowledge was obtained from the databases and literature described in Knowledge graph section, following the processes summarized in Fig. 3.

First, we performed a general literature review to collect relevant articles describing the heart muscle structure and its contraction process, the electrical conduction system of the heart, and the aorta structure and relevant biological processes. Second, we identified in the selected literature (see Table 2) the proteins that are relevant in the context of cardiomyopathies, channelopathies and aortic diseases, which are those proteins that have a relevant role in the structure or normal function of the heart or the aorta. Then, we obtain the information about the relevant proteins and the genes that codify them using Uniprot and NCBI Gene, respectively. Finally, using KEGG and Reactome, we identify the cardiac pathways and biological reactions in which these proteins participate.

**Fig. 4:**
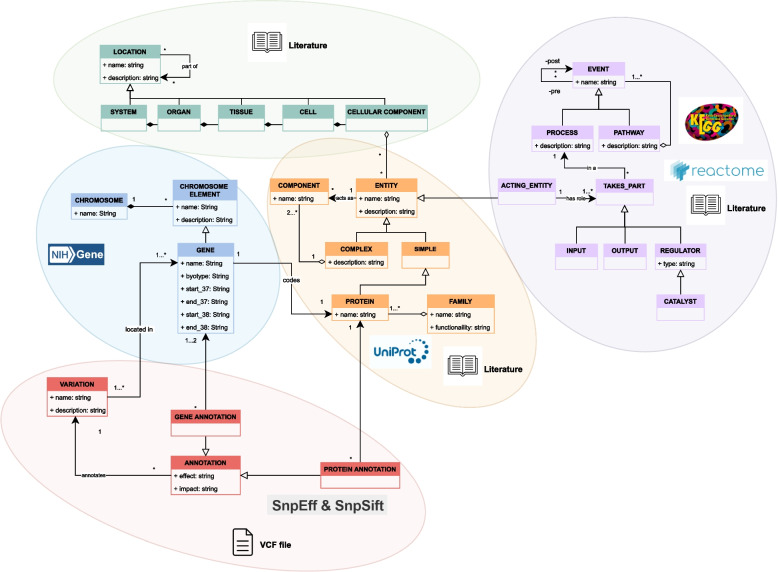
Datasets used to populate the CardioGraph data model in each of the model’s dimension

All this information was used to populate each of the dimensions of the CardioGraph data model, as Fig. 4 represents. For the instantiation of the Location dimension, none of the databases considered provided information about the heart or the aorta, the central body locations associated with familiar cardiopathies. Therefore, this dimension was instantiated only using the knowledge obtained from the literature. For the instantiation of the Structural dimension, we focused on the NCBI Gene database, as it provides all the required information about genes. Regarding the Entity dimension, the relevant proteins and other components were identified using literature, while the detailed information about each identified element was obtained mainly from Uniprot and complemented with literature when necessary. The pathway dimension was instantiated using the information provided by both Reactome and KEGG, and it was complemented with literature when necessary. Finally, the Variation dimension is a particular case. Here the information is not obtained from a database or literature. Instead, this dimension is instantiated each time a VCF variation is detected and analyzed by CardioGraph. On the one hand, the information about the variations (*Variation* class) is obtained from the VCF file itself. On the other hand, the information about the gene and protein consequences (*Gene Annotation* and *Protein Annotation* classes, respectively) is obtained with the SnpEff tool.

This instantiation process resulted in one knowledge graph per type of familiar cardiopathy. Table 3 summarizes the number of class instances performed in each knowledge graph grouped by the dimensions of the CardioGraph data model.

**Table 3:** The Number of nodes per group in Cardiomyopathies, Channelopathies and Aortic disorders in the Neo4J database

Model dimension	Cardiomyopathies	Channelopathies	Aortic disorders
Structural dimension	24	31	33
Entity dimension	35	30	20
Location dimension	13	13	9
Pathway dimension	26	46	42

The resulting knowledge graphs are too extensive to be presented in detail here. Instead, to illustrate the knowledge generated, we present one specific example of instantiation per each dimension of the CardioGraph data model in the following subsections.

#### The location dimension

**Fig. 5:**
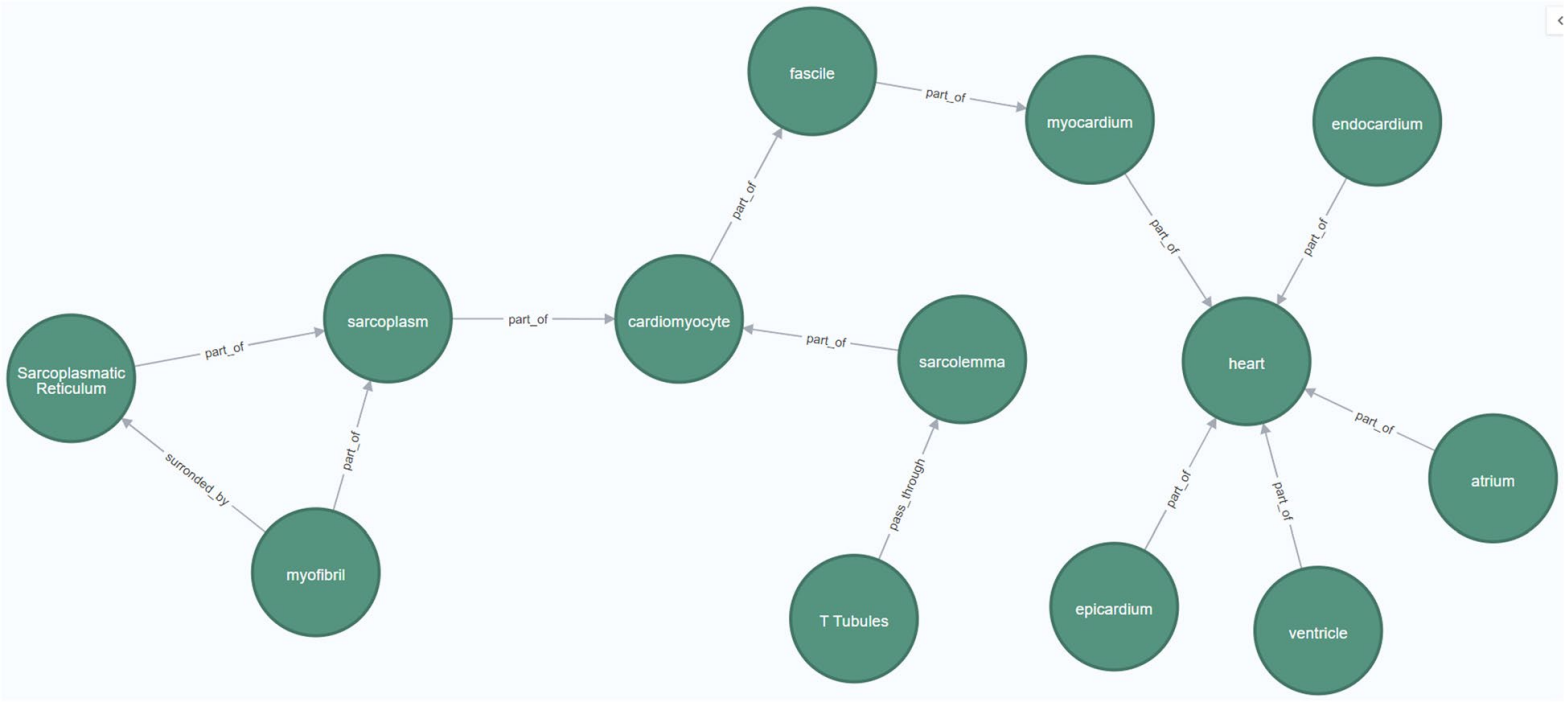
Instantation of the location dimension of the CSC

Figure 5 represents the instantiation of the Location dimension of the CardioGraph data model to represent the most essential components of the heart, the body structure affected in cardiomyopathies and channelopathies.

The instantiation begins with the representation of the *heart*. Then, we focused on the morphological structure of the heart by representing its chambers (*atria* and *ventricles* [24]), and its tissue layers (*epicardium*, the *myocardium*, and the *endocardium*).

The myocardium is the most affected tissue layer. It is organized in *fascicles*, a bundle of cells enveloped together [25]. The cells that constitute the fascicles are specialized cardiac muscle cells called *cardiomyocytes*, which are responsible for the contractile capabilities of the heart [26]. The most interesting components of the cardiomyocytes are the *sarcolemma* (e.g., cellular membrane) and the *sarcoplasm* (e.g., cytoplasm). Two important structures constitute the sarcoplasm: the *sarcoplasmatic reticulum*, responsible for providing the calcium that is needed in the heart’s contraction process [24, 25], and the *myofibrils*, the main actors of the heart’s contraction process.

#### The entity dimension

**Fig. 6:**
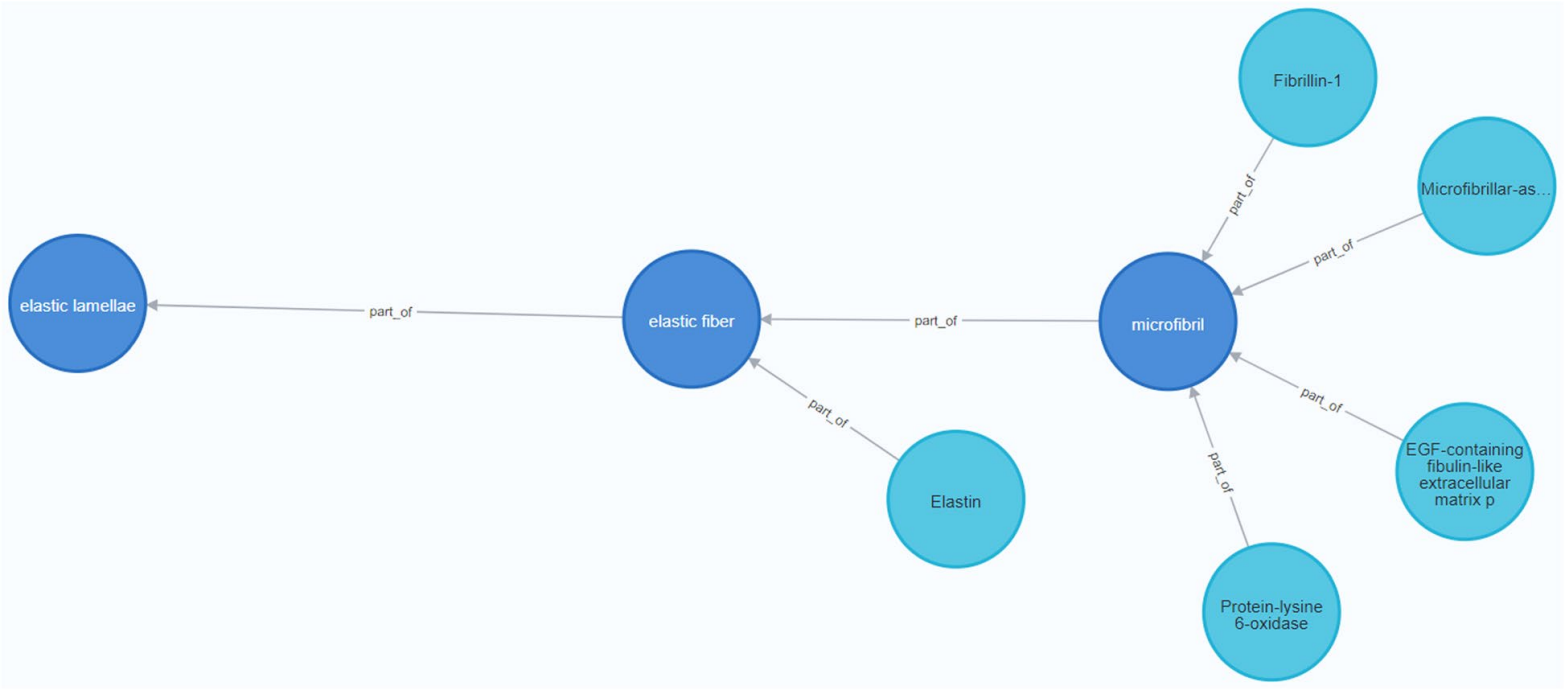
Instantiation of the entity dimension of the CardioGraph data model

Figure 6 represents the instantiation of the Entity dimension of the CardioGraph data model to represent the *Elastic Lamella*, a protein complex with high relevance in genetic aortic disorders.

The Elastic Lamella aggregates in concentric layers *Elastic fibers*, the main responsible for the stretching capabilities of the aorta [27]. The Elastic fibers are primarily constituted by the *Elastin* protein, which provides resilience and elasticity to the aorta [28]. Finally, Elastin is surrounded by elastic *microfibrils*, responsible for the Elastic fiber orientation [29].

On the other hand, the main component of *microfibrils* is the *Fibrillin* protein. Other proteins, such as *EGF-containing fibulin-like extracellular matrix protein 2*, *Protein-lysine 6-oxidase*, and *Microfibrillar-associated protein 5* bind or interact with *Fibrillin* [30].

#### The structural dimension

**Fig. 7:**
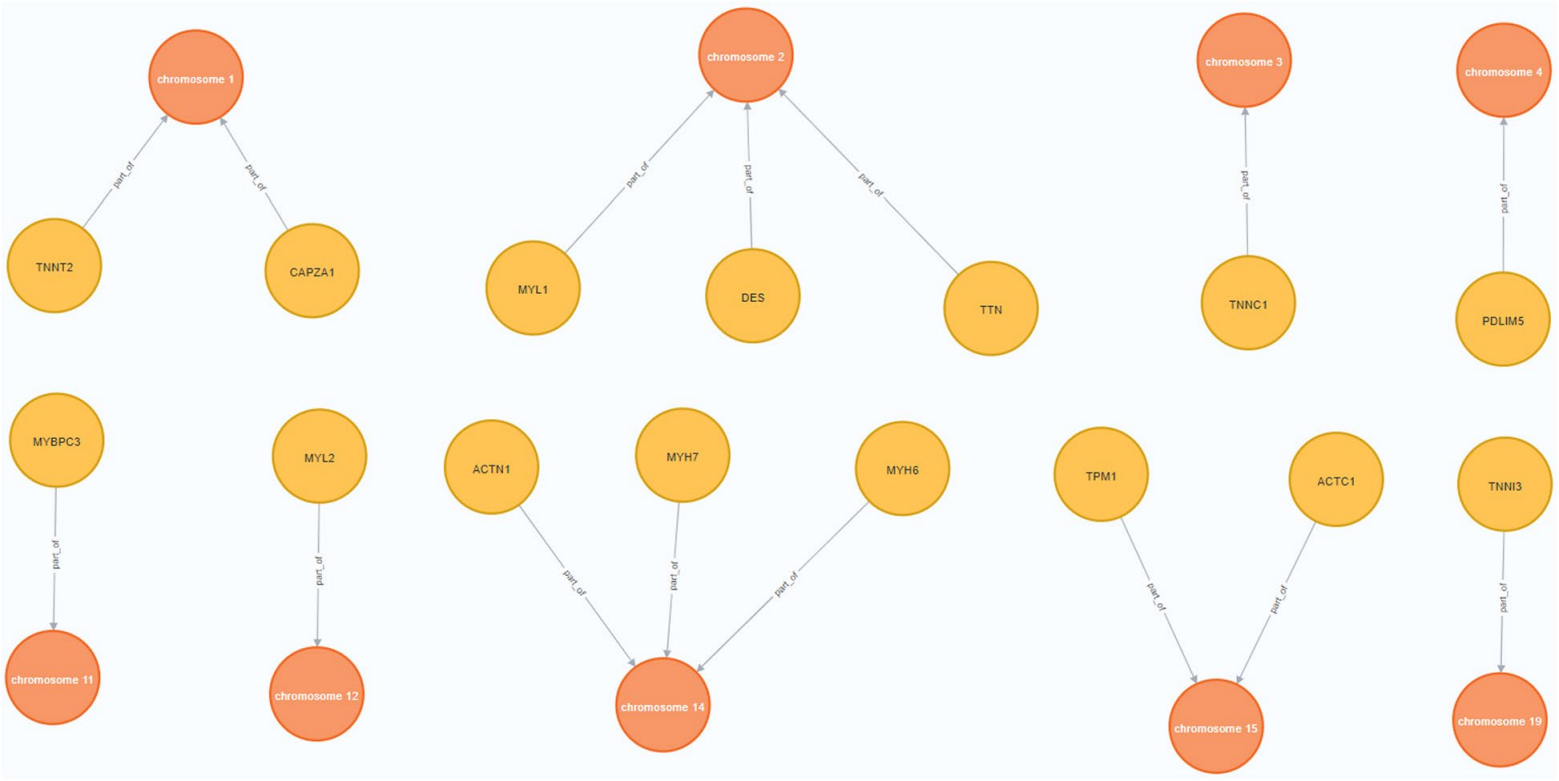
Instantiation of the structural dimension of the CardioGraph data model

Figure 7 represents the instantiation of the Structural dimension of the CardioGraph data model to represent the genes that codify for proteins involved in Cardiomyopathies as well as the chromosomes where those genes are located.

The *chromosome 1* contains the *TNNT2* and *CAPZA1* genes. The *chromosome 2* contains the *MYL1*, *DES*, and *TTN*. The *chromosome 14* contains the *ACTN1*, *MYH7*, and *MYH6* genes. The *chromosome 15* contains the *TPM1* and *ACTC1* genes. Finally, the *chromosome 3*, *chromosme 11*, and *chromosome 12*, contain the *TNNC1*, *MYBPC3*, and *MYL2* genes, respectively.

#### The pathway dimension

**Fig. 8:**
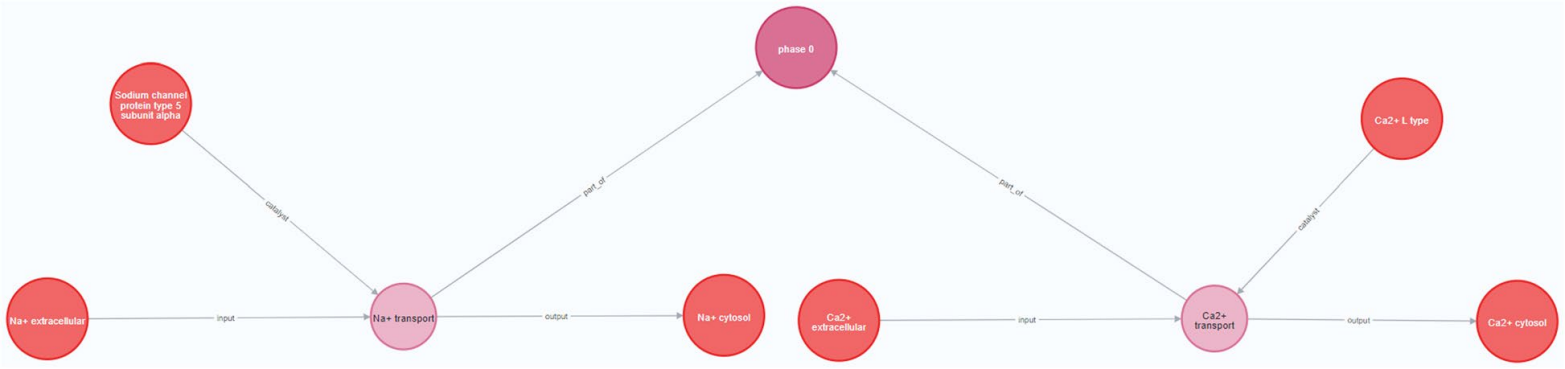
Instantiation of the pathway dimension of the CardioGraph data model

Figure 8 represents the instantiation of the Pathway dimension of the CardioGraph data model to represent the processes and entities participating in the *phase 0* pathway. This pathway is responsible for the rapid depolarization of the cardiomyocyte cells, a process vital for the correct propagation of the electrical signal in the heart’s contraction process [31]. As this pathway is directly related to the heart’s electrical activity, its malfunctioning is associated with channelopathies.

The pathway comprises two main reactions: i) *Na+ transport*, and ii) *Ca2+ transport*. On the one hand, in the *Na+ transport* process, the *Sodium channel protein type 5 subunit alfa* protein transports *Na+ ions* from the extracellular medium to the cytosol. On the other hand, in the *Ca+ transport* process, the *Ca2+ channel, L type* protein transports *Ca2+ ions* from the extracellular medium to the cytosol.

#### The variation dimension

CardioGraph instantiates the Variation dimension every time a variation from a VCF file is annotated in the server. However, this instantiation is not persistent, meaning that the variations are not permanently stored in the database. Instead, this temporary instantiation is used to present the VCF analysis results in figures and tables to the user.

### CardioGraph platform

CardioGraph follows a three-tier architecture composed of a database, a backend, and a frontend. The following subsections explain each tier in detail.

#### The database

The first tier is CardioGraph’s database. As mentioned in The platform section, Neo4J was selected as the database management system. There are three main reasons why we chose Neo4J as our database management system. The first reason is that Neo4j enabled us to represent complex and highly interconnected biological data in a natural and intuitive manner. The biological entities we are interested in, such as genes, proteins, or processes, can be efficiently represented as nodes, and their relationships can be described as edges connecting these nodes. For example, we represent biological pathways associated with heart contraction processes and the proteins that participate in those processes as a directed cyclic graph network, which is very intuitive and straightforward. The second reason is associated with the data querying capabilities of Neo4J. Here, graph-based databases have an inherent advantage over their relational counterpart, where the complex queries required to generate our insights would have required complex join operations that are much simpler using a graph data model. The ability of Cypher, a declarative query language used in neo4J databases, for seamless traversal and pattern matching in the graph allowed us to navigate over a non-defined number of edges, thus simplifying the queries over our biological data.

One of the most common visualizations in genomics is graph visualization. Because the data is complex and interconnected, several tools focus on this paradigm (e.g., Blast2GO [32], PhyD3 [33], Pangenome Graphs [34], or DisGeNET [35]). The structure of a graph intuitively represents complex relationships and dependencies between biological entities, making it easier to identify the most relevant components and their connections. Furthermore, graph visualizations aid in understanding the context and significance of individual entities within a larger network. One of CardioGraph’s primary goals is to provide valuable insights into the underlying biological processes associated with cardiopathies through this context-rich representation.

The information generated with the data model instantiation step (see CardioGraph knowledge graph section) was transformed into a graph schema equivalent to the CardioGraph data model by using a set of python scripts.

#### The backend

The second tier is CardioGraph’s backend. As mentioned in The platform section, it has been developed in JavaScript with the Node.js runtime environment. The backend consists of the following modules:

- The transport module: This module is responsible for communicating with the frontend. It implements the Application Programming Interface (API) and triggers the orchestrator module when a new request arrives.
- The orchestrator module: This module manages the other modules when an incoming request arrives, including the JavaScript and bash processes.
- The spawn module: This module is responsible for spawning new processes, including JavaScript and bash. It spawns new processes based on the orchestrator module requests.
- The queue module: This module handles the different incoming requests from the transport module.
- The file system module: This module uploads the input VCF file into the system and handles the different temporary files generated during the discovery process.
- The discovery module: This module encapsulates the logic of the discovery process (i.e., the annotation and filtering of variations and the generation of the results). This module implements the following functionalities:
  - Initializer: It creates the required external connections and initializes the data objects used in the process.
  - Parser: It parses the VCF files into a model passed as an additional parameter.
  - Filter: It filters the DNA variations of the VCF file. Only those variations located in the genes associated with cardiopathy disorders are considered.
  - Annotate: It annotates the DNA variations of the VCF file. The annotation is made using the SnpEff tool with the following commands:-Xmx8g, -noLog, -noStats, -canon, and -nextProt.
  - Insights: It generates the results of the VCF file’s DNA variations. This information is divided into three parts: aggregated, variation-specific, and additional information for relevant variations. Aggregated information includes the number of variations grouped by chromosome, gene, predicted impact, and predicted effect. Variation-specific information includes, for each variation, its chromosome, position, ref and alt alleles, affected gene and transcript(s), predicted effect of the variation, sequencing quality information, and patient-level information. Finally, those variations that are considered relevant include the specific criteria that they meet in order to be considered relevant.
- The auxiliary module: This module contains auxiliary functions required for the other modules’ correct functioning.

**Fig. 9:**
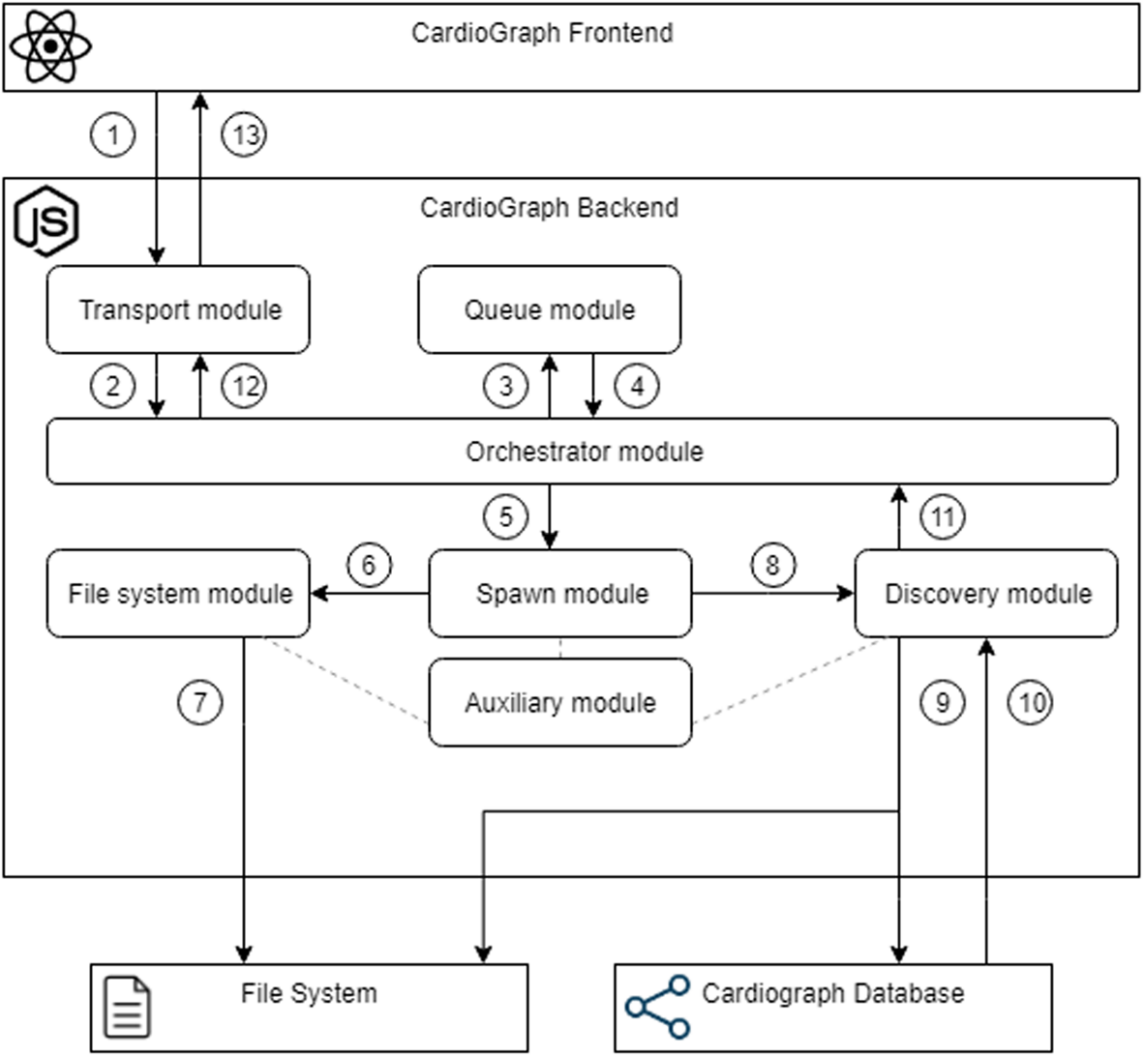
A simplified representation of the backend’s workflow of CardioGraph

Figure 9 shows a simplified view of the backend’s workflow. First, the transport module processes the incoming request and initiates the entire process (see steps 1 and 2). The orchestrator interrogates the queue module to check for any available core to spawn the other processes (see step 3). The queue alerts the orchestrator to launch a file system process that saves the received VCF file to the server file system (steps 4, 5, 6, and 7). The discovery module is then run by another process in order to generate the insights associated with the VCF file using the Neo4J database (steps 8, 9, and 10). Finally, when the discovery module completes, the orchestrator is notified, allowing the transport module to send the response to the frontend (steps 11, 12, and 13).

#### The frontend

The third tier is CardioGraph’s fronted. As mentioned in The platform section, it has been developed in JavaScript with React.js library. The frontend of the platform is divided into four sections:

- Home: This is the main section. It contains basic information about the platform and our work.
- Data Model: The data model we based our data transformation processes on is presented in this section.
- Knowledge Graph: For each type of familiar cardiopathy (i.e., genetic aortic diseases, channelopathies, and cardiomyopathies), it shows an interactive graph displaying the results of the data model instantiation for that type. Users can zoom in and out, move, and click on each graph node to show additional information.
- Discover: The user can upload a VCF file to analyze in this section. The results are displayed in six sections:
  - A summary containing the list of variations (see excerpt 1 in Fig. 1).
  - A list of relevant variations according to the set of predefined rules.
  - Two charts representing the consequences of the identified variations (see excerpt 3 in Fig. 1).
  - The distribution of variations among the different regions of the genome (see excerpt 4 in Fig. 1).
  - A grid containing all the variations with their associated information (see excerpt 5 in Fig. 1).
  - A button to download the generated insights (see excerpt 6 in Fig. 1).

**Fig. 10:**
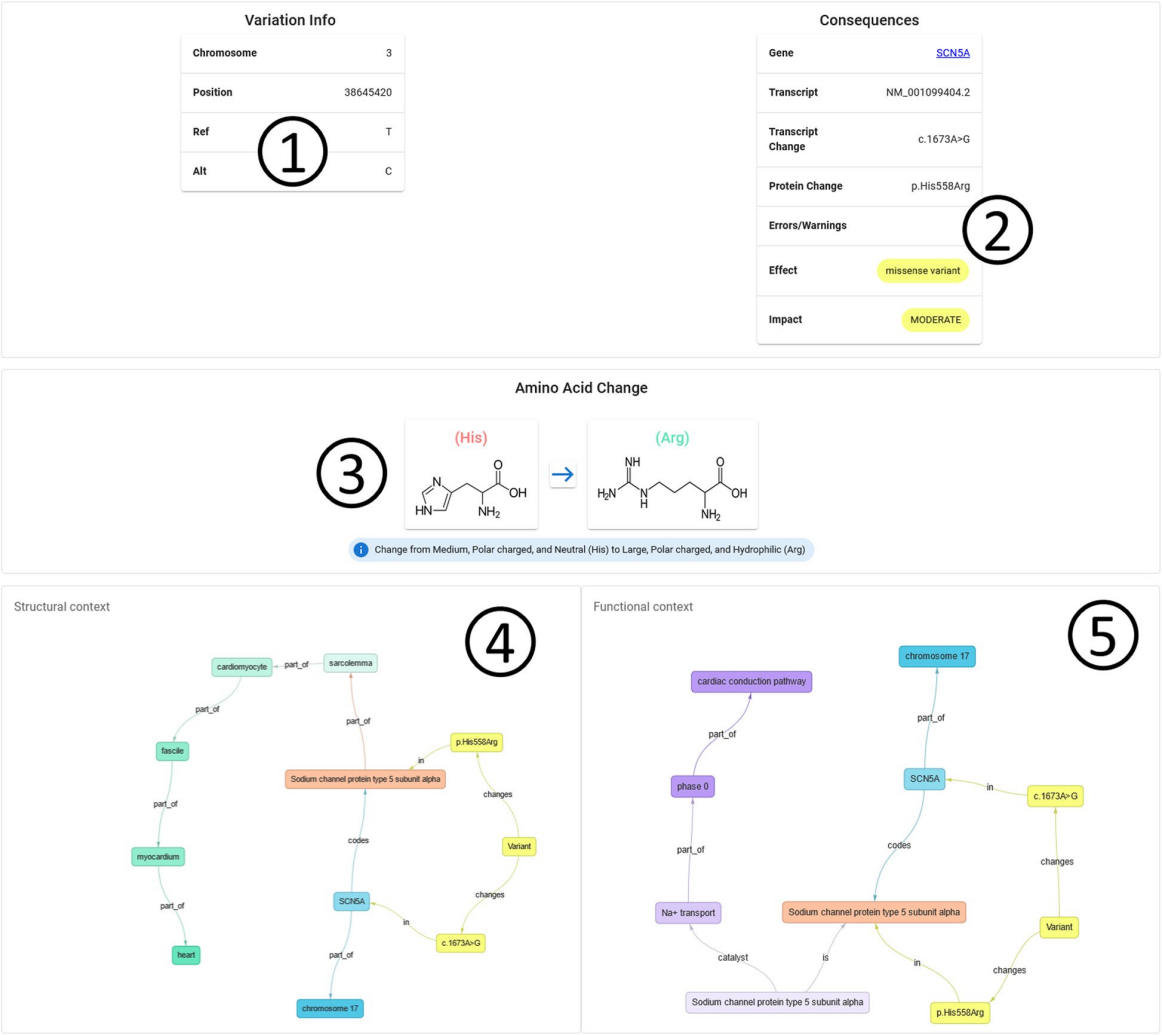
User interface displayed when showing the details of a specific variation. 1: basic information of the variation, including its position and alleles. 2: the consequence of the variation, including the affected gene, the affected transcript, and the specific effect. 3: the specific amino acid change (if any) that the variation caused. 4: the structural elements of the heart that are altered because of the variation. 5: the pathway and its specific processes that are altered because of the variation

From the Discover page, users can navigate to the specific details of a variation or a gene. For each variation, we show the following information:

1. Basic information of the variation, including its position and alleles (see excerpt 1 in Fig. 10).
2. The consequence of the variation, including the affected gene, the affected transcript, and the specific effect (see excerpt 2 in Fig. 10).
3. The specific amino acid change (if any) that the variation caused (see excerpt 3 in Fig. 10).
4. The structural elements of the heart that are altered because of the variation, i.e., structural context (see excerpt 4 in Fig. 10).
5. The pathway and its specific processes that are altered because of the variation, i.e., functional context (see excerpt 5 in Fig. 10).

**Fig. 11:**
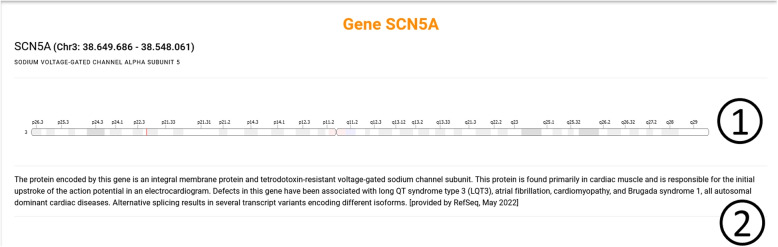
User interface displayed when showing the details of a gene. 1: the location of the gene among the chromosome. 2: a description from the gene

Finally, users can navigate to a section containing information of the affected gene from both the discover and variation page. This page displays the gene’s location in the chromosome (see excerpt 1 in Fig. 11) and a description of the gene (see excerpt 2 in Fig. 11).

## Use case

In this section, we evaluate the knowledge that can be obtained using CardioGraph. To carry out this evaluation, we defined two use cases. In the first use case, we used the ClinVar open dataset to validate the results provided by Cardiograph analyzing variations with a reported clinical significance. In the second use case, we used CardioGraph to identify relevant variations in 23 patients diagnosed with a familiar cardiopathy disorder and discuss the consistency of the results.

### Use case 1: evaluation using ClinVar

We first used CardioGraph to analyze the ClinVar dataset [13], a well-known data source that provides information about variations and their role in disease development. This dataset, in particular, contains information about the classification of variations for a disease obtained from various laboratories and clinical experts.

The most recent VCF version of ClinVar available was used for the evaluation. In particular, it was published on 2023-08-20 in the following link: https://ftp.ncbi.nlm.nih.gov/pub/clinvar/vcf_GRCh37/clinvar.vcf.gz. To keep the focus on familiar cardiopathies, ClinVar’s VCF was filtered to include only variations with classifications for either cardiomyopathy, long QT syndrome, or Marfan syndrome. Furthermore, only variations with a known effect on disease development were chosen (i.e., those classified as benign, likely benign, likely pathogenic, or pathogenic), avoiding VUS and variations with conflicting classifications.

**Fig. 12:**
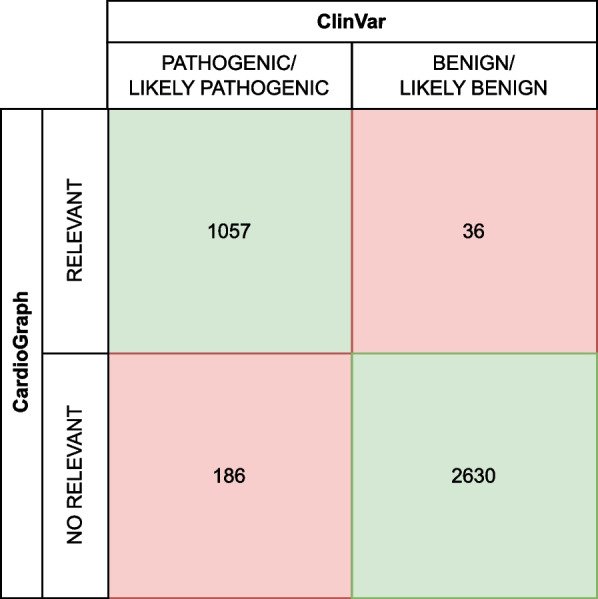
Confusion matrix

CardioGraph was used to analyze the filtered VCF from ClinVar, and the concordance between the CardioGraph results and the ClinVar classifications was examined. If ClinVar classifies the variation as benign or likely benign and CardioGraph considers the variation irrelevant, or if ClinVar classifies the variation as pathogenic or likely pathogenic and CardioGraph considers the variation relevant, we considered the results concordant. The results obtained are summarized in the confusion matrix shown in Fig. 12.

For the relevant variations, the results are concordant in 1,057 out of 1,093 cases. In the case of no relevant variations, the results are concordant in 2,630 of 2,816 cases. Assuming that the ClinVar classifications, performed by clinical experts, are the correct ones, we can draw the following conclusions:

- CardioGraph has a *sensitivity* of 85.5%, meaning it correctly identifies 85.5% of pathogenic or likely pathogenic variations in the filtered ClinVar VCF.
- CardioGraph has a *specificity* of 98.65%, meaning it correctly identifies 98.65% of benign or likely benign variations in the filtered ClinVar VCF.
- Overall, the *accuracy* of CardioGraph is 94.32%.

CardioGraph misclassified 222 variations. We investigated these variations further to determine what was causing this discrepancy. We concentrated on variations that were classified using specific assertion criteria. Note that, in ClinVar, users can submit a variation-phenotype association without specifying the assertion criteria they used. This led to a total of 21 variations classified as pathogenic or likely pathogenic and 34 as benign or likely benign.

**Fig. 13:**
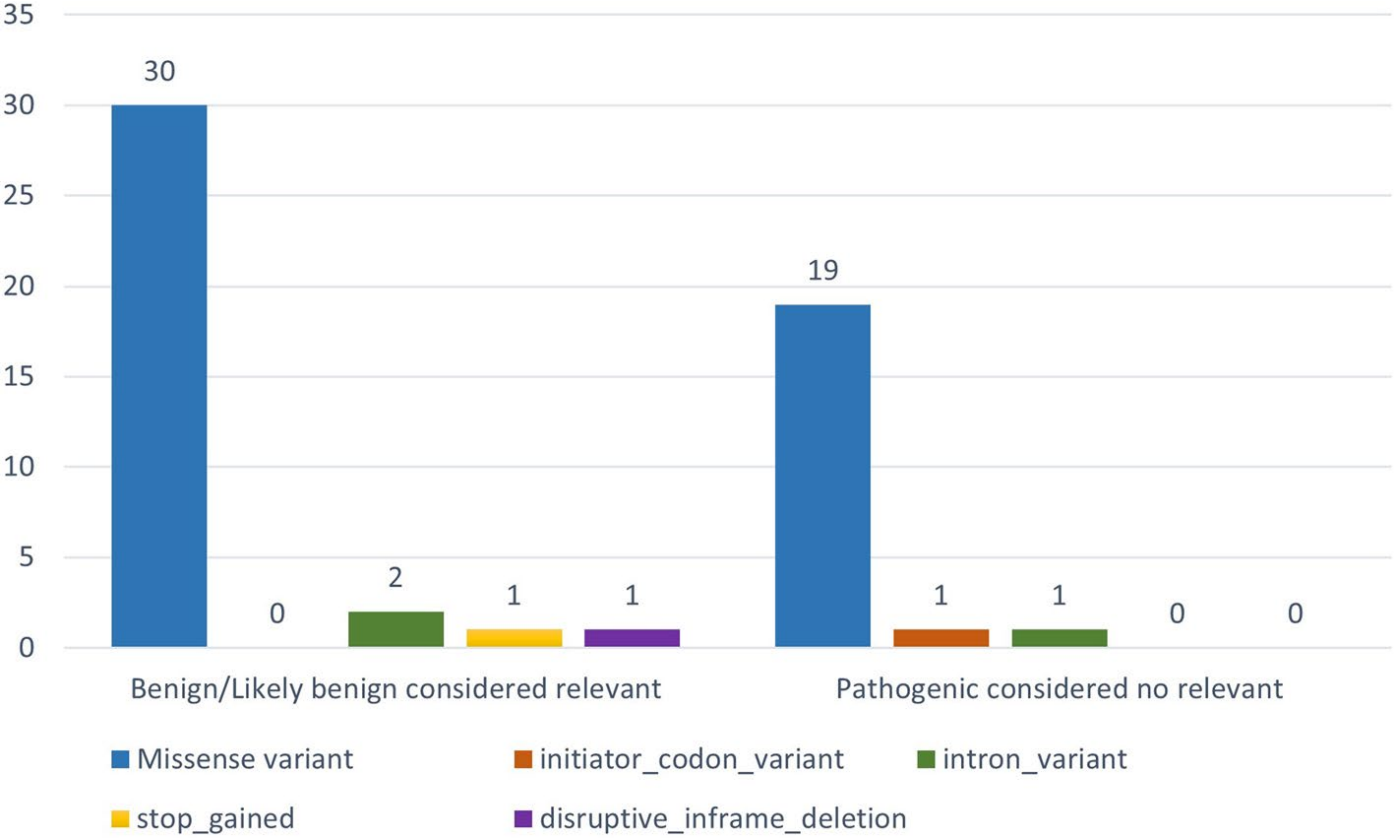
Discordance by variation type

The discordance is primarily focused on cardiomyopathy disorders (54.55%), which may be due to the broader spectrum of phenotypes that this term encompasses when compared to the other phenotypes analyzed. Furthermore, we discovered that most variations with discordant results are missense variations (see Fig. 13). Three intronic variations produce discordant results. However, since CardioGraph’s primary focus is on variations that significantly impact proteins and their related pathways, these three intronic variations will not be investigated further.

We first focused on variations identified as relevant by CardioGraph and classified as benign/likely benign by ClinVar. CardioGraph deemed all of these missense variations (see blue bar on the left-hand chart in Fig. 13) to be relevant due to the critical amino acid change they produce. Another discordant variation results in a stop gained (see yellow bar on the left-hand chart in Fig. 13), which has been deemed significant because it is predicted to result in a complete LOF and an NMD process in the protein. The remaining, disruptive inframe deletion (see purple bar on the left-hand chart in Fig. 13), has been considered to be relevant because is predicted to completely change the protein sequence and potentially affect is functionality.

Surprisingly, a stop-gained variation causing LOF and NMD was classified as benign or likely benign. Thus, we decided to classify all of the discordant variations using Varsome [36] an online tool that allows automatic classification of variations based on the ACMG-AMP guidelines 2015 [9], the gold standard in variation interpretation. Varsome classified 15 of the abovementioned variations as benign/likely benign, four as likely pathogenic (including the stop gain variation), and 15 as variations of uncertain significance. As a result, four variations are potentially better classified by CardioGraph than by ClinVar, while the other 15 have reasonable doubt about their relevance.

We repeated the analysis for the 21 variations deemed irrelevant by CardioGraph and pathogenic by ClinVar. In this case, most of the discordance is caused by missense variations classified as irrelevant because they do not result in a critical amino acid change (see blue bar of the right-hand chart of Fig. 13). Varsome classifies those variations as likely pathogenic but not pathogenic, with uncertain significance in one case. The initiator codon variation (see red bar of the right-hand chart of Fig. 13) was classified as Pathogenic by Varsome. We investigated further and found that Varsome, ClinVar, and other annotation tools like VEP consider this variation to produce a start loss rather than an initiator codon variation. The source of this discordance is SnpEff because it predicted a different consequence. CardioGraph would have classified this variation as relevant because a start loss significantly impacts the protein sequence.

### Use case 2: clinical evaluation

This section reports the results of analyzing the genetic information of 23 patients that suffer from a familiar cardiopathy using Cardiograph. These patients are enrolled in the OGMIOS project (INNEST/2021/57, Valencian Innovation Agency), which intends to achieve a better understanding of the genetics of familiar cardiopathies and providing clinical experts with the tools to transfer genetic knowledge to real clinical practice. As Table 4 shows, 13 patients suffered from a cardiomyopathy, while 10 suffered from a channelopathy. No patients with a genetic aortic disorder were available for this study.

**Table 4:** Patients studied per group of disorder

Group of disorder	Disorder	Patient IDs
Cardiomyopathy	Dilated Cardiomyopathy	03-002, 03-003, 03-009, 03-012, 03-014, 03-018, 03-019
	Hypertrophic Cardiomyopathy	03-005, 03-006, 03-008, 03-013, 03-016, 03-020
Channelopathy	Long QT Syndrome	03-001, 03-004, 03-011, 03-015, 03-017, 03-029, 03-030, 04-032, 04-033, 04-034

**Fig. 14:**
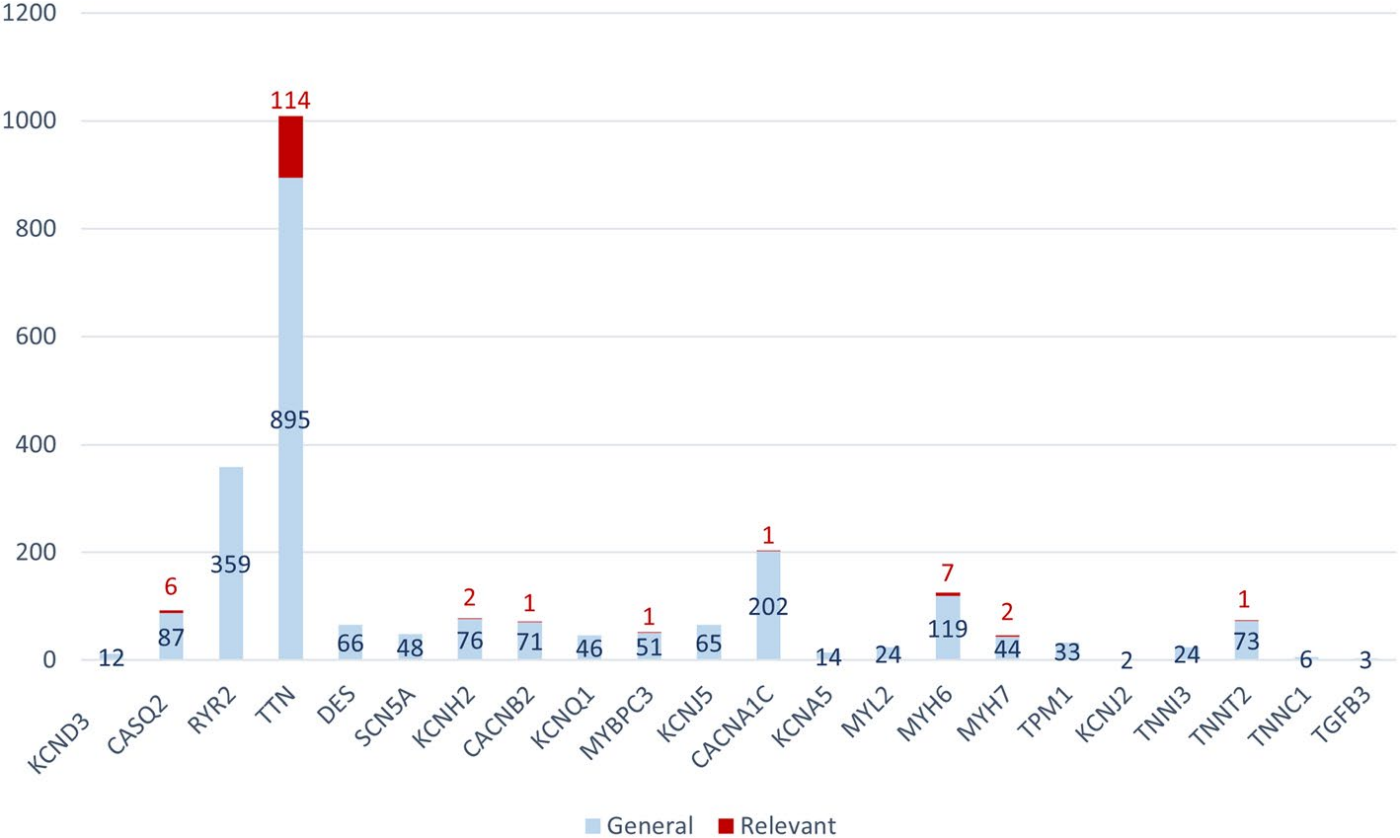
Distribution of variations per gene in patients with cardiomyopathy disorders. In blue, all variations detected by CardioGraph. In red, those variations that considered relevant by CardioGraph

Figure 14 shows the distribution of variations per gene for all the patients with a cardiomyopathy. Relevant variations are depicted in red, while those not considered relevant are depicted in blue.

Most of the variations and relevant variations are located in the TTN gene, which codes for protein that has a structural role in the cardiomyocyte cells of the heart muscle (see Fig. 5). The remaining relevant variations are associated with genes with a vital role in the heart’s muscle contraction (i.e., MYBPC3, MYH6, MYH7, and TNNT2).

The genes associated with the heart’s electrical activity are also affected in some of the patients that suffer from cardiomyopathy. On the one hand, the CACNB2, CASQ2, and CACNA1C genes have an essential role in Ca2+ ion transport, which is important for the rapid depolarization that occurs in the first pathway (phase 0). This pathway is involved in the action potential of the heart’s cells that regulates the heart’s contraction rhythm. On the other hand, the KCNH2 gene participates in the K+ transport, which is part of the rapid repolarization pathway (phase 3) of the heart’s action potential. These reactions are usually associated with channelopathies instead of cardiomyopathies. However, CardioGraph identified that some patients suffering from cardiomyopathy may have altered not only the structure of the heart but also some of its electrical processes.

**Fig. 15:**
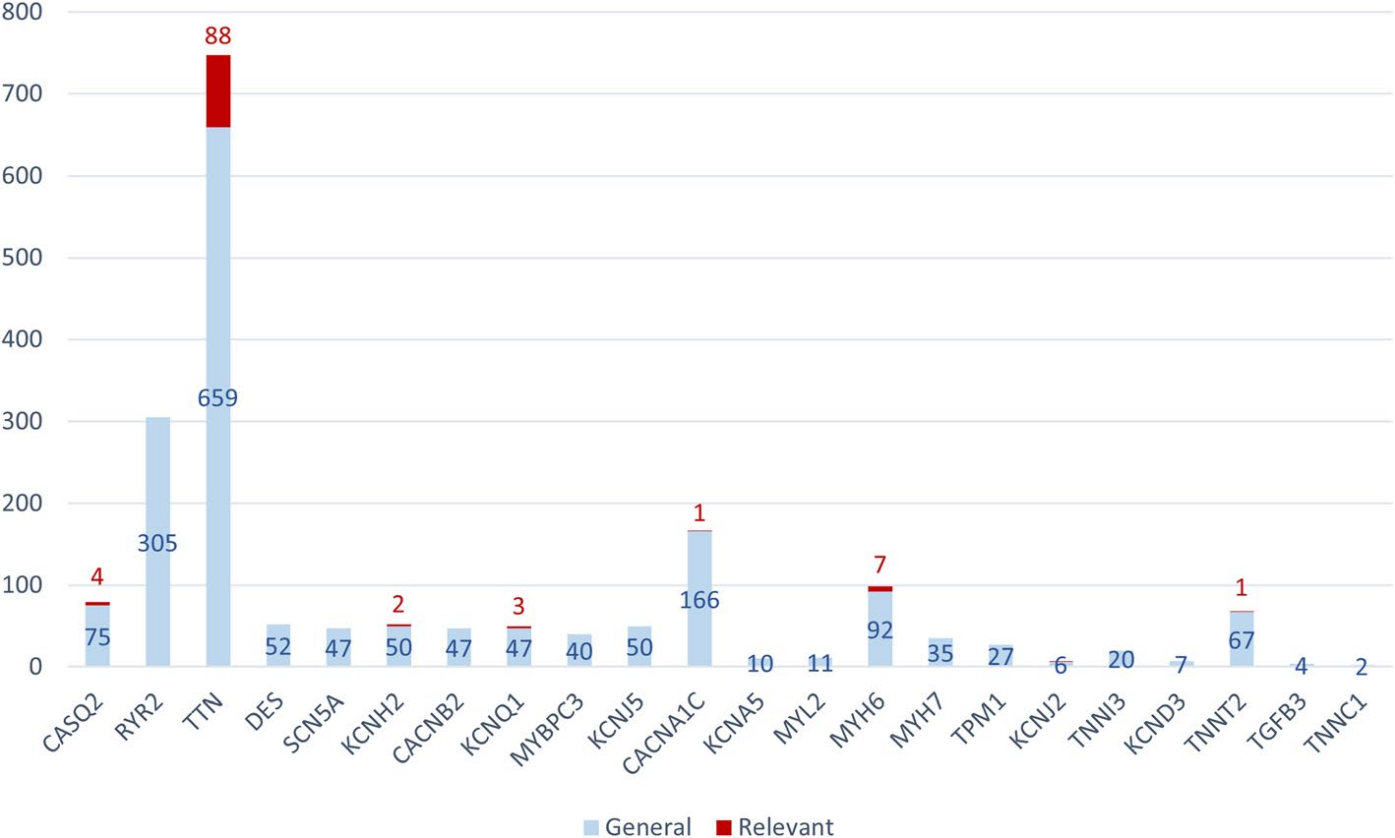
Distribution of variations per gene in patients with channelopathy disorders

Figure 15 shows the distribution of variations per gene for all the patients suffering from a channelopathy disorder. Again, relevant variations are depicted in red, while those not considered relevant are depicted in blue.

Like with cardiomypathies, the TTN gene is the most affected one by relevant variations, followed by the MYH6 gene. Both genes have important roles on the heart’s structure and contraction process, which is not expected to be affected in channelopathies. Again, CardioGraph has detected these somewhat unexpected potential alterations. Besides, the CASQ2, CACNA1C, and KCNH2 genes are affected by relevant variations, which means that the channelopathy patients present alterations in the phase 0 or the phase 3 pathways of the heart’s electrical activity. The KCNQ1 gene, which is involved in the K+ transport in the phase 3 pathway, is also affected.

CardioGraph allowed us to efficiently interpret the genetic information stored in the VCF files of patients and identified unexpected associations. We could determine the parts of the heart affected and the exact functionalities probably altered or damaged in all the patients we studied. Thus, CardioGraph has the potential to provide valuable information for diagnosing patients with familiar cardiopathies.

## Discussion

### Cardiograph

CardioGraph is a tool specifically designed for variation prioritization in the context of familiar cardiopathies. In our evaluation using a benchmark dataset, CardioGraph was able to distinguish between those variations that are potentially pathogenic for familiar cardiopathies and those that do not cause disease with an accuracy of 94.32% (see Use case 1: evaluation using ClinVar section). However, we also identified some issues that could improve the accuracy of CardioGraph when addressed. First, most prioritization errors occur when analyzing missense variations that do not result in a critical amino acid change. To improve the analysis of such variations, checking whether the variation is located in a mutational hotspot or a relevant protein domain could improve the evaluation of these variations.

The second issue we discovered is that up to 50% of the incorrectly considered relevant variations are found in a TTN gene. The TTN gene is notable for its length, as it is the longest gene in humans that codes for a protein. This gene has 364 exons and produces an RNA that is more than 100 kb long. As a result, this gene is expected to contain a more significant number of variations, potentially increasing the number of false positive results. Consequently, during the variation prioritization process, it is required to consider the particularities of each gene. For instance, truncating variations in the TTN gene are known to be the most frequent type of variations causing dilated cardiomyopathy rather than missense variations.

### Other relevant tools

From a more technological perspective, Related work section describes nine widely known tools that offer some functionality that matches that offered by CardioGraph. We now analyze similarities and differences between CardioGraph and these tools regarding their capabilities. More specifically, we focused on three categories: information representation, output, and variation-specific output for those offering such functionality.

Table 5 summarizes the results of our analysis in terms of what information is represented and how. CardioGraph and CardioClassifier are the only platforms allows for uploading and analyzing a VCF file. CardioGraph is the only platform that describes the underlying model used to structure, describe, and manage the information. Having a well-defined data model can help in terms of scalability, maintainability, and knowledge generation [37]. An important feature in which CardioGraph surpasses the others is that it analyzes the three known types of familiar cardiopathies (genetic aortic disorders, channelopathies and cardiomyopathies), while the other platforms only consider one.

**Table 5:** The list of general characteristics of the tools analyzed

Tool	VCF input	Data model	Disease information		
			Aorthopaties	Channelopathies	Cardiomyopathies
ACGV	$\times$	$\times$	$\times$	$\times$	✓
CardioClassifier	✓	$\times$	$\times$	$\times$	✓
Marfan Syndrome (FBN1)	$\times$	$\times$	✓	$\times$	$\times$
TTN database	$\times$	$\times$	$\times$	$\times$	✓
HCM risk predictor	$\times$	$\times$	$\times$	$\times$	$\times$
ARVC database	$\times$	$\times$	$\times$	$\times$	$\times$
PhenoDis	$\times$	$\times$	$\times$	$\times$	✓
CardioGraph	✓	✓	✓	✓	✓

Table 6 summarizes the results of our analysis in terms of the output they provide. The following four characteristics have been analyzed:

1. **Highlight the relevant information**: CardioGraph, ACGV, CardioClassifier, and the TTN database can prioritize information and highlight what they consider relevant. All the platforms but CardioGraph offer such information by means of tables, whereas CardioGraph displays different charts.
2. **Provide variation-specific information**: All of the analyzed platforms provide variation-specific information except for the HCM risk predictor and PhenoDis. Instead, they provide a general summary of the variations without delving into specific details about any of them.
3. **Provide gene-specific information**: Only four platforms show information associated with genes. On the one hand, CardioGraph, ACGV, and CardioClassifier consider several genes associated with familiar cardiopathies. On the other hand, the TTN database only considers the TTN gene.
4. **Download the output**: Only CardioGraph, ACGV, and PhenoDis allow for downloading the generated output.

**Table 6:** Output generated by the tools analyzed

Tool	Relevant results	Variation-specific	Gene information	Download results
ACGV	✓	✓	✓	✓
CardioClassifier	✓	✓	✓	$\times$
Marfan Syndrome (FBN1)	$\times$	✓	$\times$	$\times$
TTN database	✓	✓	TTN	$\times$
HCM risk predictor	$\times$	$\times$	$\times$	$\times$
ARVC database	$\times$	✓	$\times$	$\times$
PhenoDis	$\times$	$\times$	$\times$	✓
CardioGraph	✓	✓	✓	✓

Table 7 summarizes the results of our analysis in terms of variation-specific information they provide. There are six platforms that retrieve variation-specific information: ACGV, CardioGraph, CardioClassifier, Marfan Syndrome (FBN1), TTN database, and ARCV. We considered the following three characteristics:

1. **The consequence of the variation**: Only CardioGraph, ACGV, and CardioClassifier provide information about the consequence of variations by reporting a high-level impact. However, CardioGraph describes the specific consequence on the transcript and the protein.
2. **The amino acid change**: CardioGraph, ACGV, and Marfan Syndrome (FBN1) are the only tools that provide information about the amino acid change produced by the variation. However, CardioGraph displays this change in a visual manner that shows the amino acid structure, its polarity, and its hydropathy. This facilitates the interpretation of the amino acid change produced by the variation compared to the ACGV, and Marfan Syndrome (FBN1) platforms.
3. **The structural and functional context**: The Marfan Syndrome (FBN1) platform only provides structural context while CardioGraph, the TTN database, and CardioClassifier describes both contexts. However, only CardioGraph represents these features visually using graphs, which facilitates the information understanding.
4. **Loss-of function (LOF) and Nonsense-mediated decay (NMD)**: Only CardioGraph provides information about LOF and NMD effects of variations.

**Table 7:** Output generated by those tools reporting variation-specific information

Tool	Variation consequences	Amino acid change	Context		LOF	NMD
			Structural	Functional		
ACGV	Effect	✓	$\times$	$\times$	$\times$	$\times$
CardioClassifier	Effect	$\times$	✓	✓	$\times$	$\times$
Marfan Syndrome (FBN1)	$\times$	✓	✓	$\times$	$\times$	$\times$
TTN database	$\times$	$\times$	✓	✓	$\times$	$\times$
ARVC database	$\times$	$\times$	$\times$	$\times$	$\times$	$\times$
CardioGraph	✓	✓	✓	✓	✓	✓

### Privacy concerns

There are several privacy concerns when sharing VCF files. Some of the most significant issues with VCF file exchange are outlined here. The first issue is personal identification as, despite the possibility of anonymizing VCF data, reidentification is still theoretically feasible. Data ownership and control pose a second challenge, as stringent controls are needed to determine who is the data’s owner and who can access and utilize it. Data encryption is the third and final concern. To protect sensitive data while moving it over networks, secure and encrypted data transmission and storage are necessary. CardioGraph must address these concerns in order to protect the confidential information included in these files.

The concerns have been addressed in CardioGraph by providing two options for use: a publicly accessible web application and a local installation. While the publicly available web application is intended for testing with small, non-critical subsets of data, the local installation allows for the tool to be used in a local, secure environment with no privacy concerns associated with the exchange of VCF files. Furthermore, the platform implements a server with HTTPS for secure and encrypted transport protocols, and the publicly accessible version of our platform regularly deletes all data uploaded.

### Competency questions

We started by facing a problem of variation identification in the context of cardiac diseases. Our first step was to learn about the domain. We used conceptual modeling to create a comprehensive representation based on four competency questions (see Data model section). Because Cardiograph is a model-based tool that strictly adheres to the data formats and connections defined in the model, we ensured that competency questions are explicitly linked to the model and that users can answer them using Cardiograph.

CardioGraph’s interface was created based on our specific model so clinical experts can quickly answer these competency questions for each variation. The model is an essential component of our solution because it is the foundation for the underlying queries and the user interface (UI). To be more specific:

Q1: **How is a gene affected by a variation?** Cardiograph identifies the specific gene that is affected by a variation, pinpoints the specific change, and provides detailed information about its impact (see Fig. 10, number 2).
Q2: **How is a protein affected by a variation?** Cardiograph describes the particular change caused by the variation and the structural and functional consequences of the variation on proteins (see Fig. 10, numbers 2 and 3).
Q3: **How is a biological pathway affected by a variation?** Cardiograph depicts a knowledge graph that depicts the biological pathways and processes that are affected by a variation in the heart structures. This enables users to gain insights into the disruption or modulation of cardiac-associated pathways (see Fig. 10, number 4.)
Q4: **How is a particular heart biological element affected by a variation?** Cardiograph depicts a knowledge graph that connects a variation to the affected heart structures, which include cells, tissues, and more complex structures (see Fig. 10, number 5.)

Cardiograph streamlines data analysis and interpretation with a combination of an intuitive UI and automated discovery processes. The tool reduces user workload by providing a user-friendly platform that allows for simple navigation through complex data. In addition, Cardiograph automates the discovery of potentially clinically relevant variations, thereby saving time for data interpretation.

## Conclusions

We have presented CardioGraph, a platform that supports the analysis of novel variations associated with familiar cardiopathies. This platform annotates the variations of a VCF file and can prioritize them. It also provides information about the variations’ effect at the transcript and protein levels, providing each variation’s functional and structural context.

Since most of the studied variations in familiar cardiopathies novel, CardioGraph has the potential to help clinicians deliver more accurate genomic-based diagnoses to their patients.

Future work is oriented in four directions. First, to expand CardioGraph’s Knwoledge Graph by including new genes and pathways associated with cardiology disorders that have not been considered in this first version. Here, one of our focus will be connecting our data with existing ontologies. Genomics Ontologies are highly relevant and widely used in the community, consisting of structured representations of knowledge consisting of concepts, relationships, and attributes. They are standardized vocabularies that allow for data standardization, integration, interoperability, and semantic understanding across different systems and communities. We have already identified ontologies of interest, as well as the model dimensions and data sources associated with each of them:

- *The location dimension*: Cell Ontology (https://www.ebi.ac.uk/ols4/ontologies/cl, BRENDA Tissue Ontology https://www.ebi.ac.uk/ols4/ontologies/bto, Gene Ontology https://www.ebi.ac.uk/ols4/ontologies/go, and Uberon https://www.ebi.ac.uk/ols4/ontologies/uberon.
- *The Entity dimension*: Gene Ontology and Protein Ontology https://www.ebi.ac.uk/ols4/ontologies/pr.
- *The Structural dimension*: Gene Ontology and Sequence Ontology https://www.ebi.ac.uk/ols4/ontologies/so.
- *The Pathway dimension*: Pathway Ontology https://www.ebi.ac.uk/ols4/ontologies/pw and Protein Ontology.
- *The Variation dimension*: Sequence Ontology and Variation Ontology https://www.ebi.ac.uk/ols/ontologies/vario.

Finally, the two remaining dimensions of our future work will include both additional information of interest to assess the relevance of variations (e.g., functional studies or hotspots) and new data sources with information about previously reported variations to complement novel variations with those already reported.

## Data Availability

Not applicable.

## Abbreviations

ACGV: Atlas of cardiac genetic variation
ACMG/AMP: American college of medical genomics / association for molecular pathology
ACTC1: Cardiac muscle alpha actin
ACTN1: Alpha actinin 1
ADP: Adenosine diphosphate
API: Application programming interface
ARVC: Arrhythmogenic right ventricular cardiomyopathy
ATP: Adenosine triphosphate
Ca: Calcium
CACNA1C: Calcium voltage-gated channel subunit alpha 1 C
CACNB2: Calcium voltage-gated channel auxiliary subunit beta 2
CAPZA1: Capping actin protein of muscle z-line subunit alpha 1
CASQ2: Calsequestrin 2
CQ: Competency question
CSC: Conceptual schema of cardiopathies
CSHG: Conceptual schema of the human genome
DCM: Dilated cardiomyopathy
DES: Desmin
DNA: Deoxyribonucleic acid
EGF: Epidermal growth factor
ExAC: Exome aggregation consortium
FBN1: Fibrilin 1
HCM: Hypertrophic cardiocyopathy
HTTPS: Hypertext transfer protocol secure
HUGO Database: Human genome organization database
ICD: Implantable cardioverter-defibrilator
ISGE Method: Identify, select, generate method
K: Potasium
KCNH2: Potassium voltage-gated channel subfamily H member 2
KEGG: Kyoto encyclopedia of genes and genomes
LOF: Loss of functionality
LOVD: Leiden open variation database
MYBPC3: Myosin binding protein C3
MYH6: Myosin heavy chain 6
MYH7: Myosin heavy chain 7
MYL1: Myosin light chain 1
MYL2: Myosin light chain 2
Na: Sodium
NAR: Nucleic acid research
NCBI: National center for biotechnology information
NMD: Nonsense-mediated mRNA decay
RNA: Ribunocleic acid
SCD: Sudden cardiac death
TNNC1: Troponin C1, slow skeletal and cardiac type
TNNT2: Troponin C2, fast skeletal type
TPM1: Tropomyosin 1
TTN: Titin
TTNT2: Troponin T2, cardiac type
UI: User interface
VCF: Variant call format
VEP: Variant effect predictor
VUS: Variant of unknown significance

## References

[1] Barriales-Villa R, Gimeno-Blanes JR, Zorio-Grima E, Ripoll-Vera T, Evangelista-Masip A, Moya-Mitjans A, et al. Plan of action for inherited cardiovascular diseases: synthesis of recommendations and action algorithms. Rev Esp Cardiol (Engl Ed). 2016;69(3):300–9. 10.1016/j.rec.2015.11.029. 26856793 10.1016/j.rec.2015.11.029

[2] Maron BJ, Maron MS, Semsarian C. Genetics of hypertrophic cardiomyopathy after 20 years. J Am Coll Cardiol. 2012;60(8):705–715. Publisher: American College of Cardiology Foundation. 10.1016/j.jacc.2012.02.068. 10.1016/j.jacc.2012.02.068 22796258

[3] García SA, et al. A conceptual model-based approach to improve the representation and management of omics data in precision medicine. IEEE Access. 2021;9:154071–85. 10.1109/ACCESS.2021.3128757.

[4] Bernasconi A, García S A, et al. A comprehensive approach for the conceptual modeling of genomic data. In: Ralyté J, Chakravarthy S, Mohania M, Jeusfeld MA, Karlapalem K, editors. Conceptual Modeling. Lecture Notes in Computer Science. Springer International Publishing; 2022. pp. 194–208. 10.1007/978-3-031-17995-2_14.

[5] Cingolani P, Platts A, Coon M, Nguyen T, Wang L, Land SJ, et al. A program for annotating and predicting the effects of single nucleotide polymorphisms, SnpEff: SNPs in the genome of Drosophila melanogaster strain w1118; iso-2; iso-3. Fly. 2012;6(2):80–92. 22728672 10.4161/fly.19695 PMC3679285

[6] Chandak P, Huang K, Zitnik M. Building a knowledge graph to enable precision medicine. Sci Data. 2023;10. 10.1038/s41597-023-01960-3. 10.1038/s41597-023-01960-3 PMC9893183 36732524

[7] Wang H, Zu Q, Lu M, Chen R, Yang Z, Gao Y, et al. Application of medical knowledge graphs in cardiology and cardiovascular medicine: a brief literature review. Adv Ther. 2022;39. 10.1007/s12325-022-02254-7. 10.1007/s12325-022-02254-7 PMC9402764 35908002

[8] Karczewski K, Weisburd B, Thomas B, Solomonson M, Ruderfer D, Kavanagh D, et al. The ExAC browser: displaying reference data information from over 60 000 exomes. Nucleic Acids Res. 2016;45. 10.1093/nar/gkw971. 10.1093/nar/gkw971 PMC5210650 27899611

[9] Richards S, Aziz N, Bale S, Bick D, Das S, Gastier-Foster J, et al. Standards and guidelines for the interpretation of sequence variants: a joint consensus recommendation of the american college of medical genetics and genomics and the association for molecular pathology. Genet Med Off J Am Coll Med Genet. 2015;17. 10.1038/gim.2015.30. 10.1038/gim.2015.30 PMC4544753 25741868

[10] Cotton R, Horaitis O. The HUGO mutation database initiative. Human genome organization. Pharmacogenomics J. 2002;2:16–9. 10.1038/sj.tpj.6500070. 11990375 10.1038/sj.tpj.6500070

[11] Maisch B, Mahrholdt H. European society of cardiology. [The 2014 ESC guidelines on the diagnosis and management of hypertrophic cardiomyopathy: what is new?]. Herz. 2014;39(8):919–30. 10.1007/s00059-014-4177-z. 25410471 10.1007/s00059-014-4177-z

[12] Fokkema IFAC, Taschner PEM, Schaafsma GCP, Celli J, Laros JFJ, den Dunnen JT. LOVD v.2.0: the next generation in gene variant databases. Hum Mutat. 2011;32(5):557–63. 10.1002/humu.21438. 21520333 10.1002/humu.21438

[13] Landrum MJ, et al. ClinVar: improving access to variant interpretations and supporting evidence. Nucleic Acids Res. 2017;46(D1):D1062–7. 10.1093/nar/gkx1153. 10.1093/nar/gkx1153 PMC5753237 29165669

[14] Kelly M, Caleshu C, Morales A, Buchan J, Wolf Z, Harrison S, et al. Adaptation and validation of the ACMG/AMP variant classification framework for MYH7-associated inherited cardiomyopathies: Recommendations by ClinGen’s Inherited Cardiomyopathy Expert Panel. Genet Med. 2018;20. 10.1038/gim.2017.218. 10.1038/gim.2017.218 PMC5876064 29300372

[15] Choi H, Pavelka N. When one and one gives more than two: challenges and opportunities of integrative omics. Front Genet. 2012;2:105. 10.3389/fgene.2011.00105. 22303399 10.3389/fgene.2011.00105 PMC3262227

[16] García S A, Casamayor JC, Pastor O. ISGE: a conceptual model-based method to correctly manage genome data. In: Nurcan S, Korthaus A, editors. Intelligent Information Systems - CAiSE Forum 2021, Melbourne, VIC, Australia, June 28 - July 2, 2021, Proceedings. vol. 424 of Lecture Notes in Business Information Processing. Cham: Springer; 2021. pp. 47–54. 10.1007/978-3-030-79108-7_6.

[17] The UniProt Consortium. UniProt: the universal protein knowledgebase in 2021. Nucleic Acids Res. 2020;49(D1):D480–9. 10.1093/nar/gkaa1100. 10.1093/nar/gkaa1100 PMC7778908 33237286

[18] Gillespie M, Jassal B, Stephan R, Milacic M, Rothfels K, Senff-Ribeiro A, et al. The reactome pathway knowledgebase 2022. Nucleic Acids Res. 2021;50(D1):D687–92. 10.1093/nar/gkab1028. 10.1093/nar/gkab1028 PMC8689983 34788843

[19] Kanehisa M, Furumichi M, Sato Y, Kawashima M, Ishiguro-Watanabe M. KEGG for taxonomy-based analysis of pathways and genomes. Nucleic Acids Res. 2022. 10.1093/nar/gkac963. 10.1093/nar/gkac963 PMC9825424 36300620

[20] Bethesda (MD): National Library of Medicine (US) NCfBI. Gene [Internet]. 2004. https://www.ncbi.nlm.nih.gov/gene/. Accessed 1 Jan 2024.

[21] McLaren W, Gil L, Hunt S, Riat H, Ritchie G, Thormann A, et al. The ensembl variant effect predictor. Genome Biol. 2016;17:1474–760X. 10.1101/042374. 10.1186/s13059-016-0974-4 PMC4893825 27268795

[22] Wang K, Li M. ANNOVAR: functional annotation of genetic variants from high-throughput sequencing data. Nucleic Acids Res. 2010;38: e164. 10.1093/nar/gkq603. 20601685 10.1093/nar/gkq603 PMC2938201

[23] Guizzardi G, Wagner G, Almeida JPA, Guizzardi RS. Towards ontological foundations for conceptual modeling: the unified foundational ontology (UFO) story. Appl Ontol. 2015;10(3–4):259–71.

[24] Hall JE, Guyton AC. Guyton and hall textbook of medical physiology. Philadelphia: Saunders/Elsevier; 2011.

[25] van der Velden J, Stienen GJM. Cardiac disorders and pathophysiology of sarcomeric proteins. Physiol Rev. 2019;99(1):381–426. 10.1152/physrev.00040.2017. 30379622 10.1152/physrev.00040.2017

[26] Woodcock EA, Matkovich SJ. Cardiomyocytes structure, function and associated pathologies. Int J Biochem Cell Biol. 2005;37(9):1746–51. 10.1016/j.biocel.2005.04.011. 15950518 10.1016/j.biocel.2005.04.011

[27] Yu X, Turcotte R, Seta F, Zhang Y. Micromechanics of elastic lamellae: unravelling the role of structural inhomogeneity in multi-scale arterial mechanics. J R Soc Interface. 2018;15(147):20180492. 10.1098/rsif.2018.0492. 30333250 10.1098/rsif.2018.0492 PMC6228495

[28] Kristensen JH, Karsdal MA. Chapter 30 - Elastin. In: Karsdal MA, editor. Biochemistry of Collagens, Laminins and Elastin. Academic Press; 2016. pp. 197–201. 10.1016/B978-0-12-809847-9.00030-1.

[29] Van den Berg F. 4.3 - Extracellular matrix. In: Schleip R, Findley TW, Chaitow L, Huijing PA, editors. Fascia: The Tensional Network of the Human Body. Oxford: Churchill Livingstone; 2012. pp. 165–70. 10.1016/B978-0-7020-3425-1.00058-1.

[30] Thomson J, Singh M, Eckersley A, Cain SA, Sherratt MJ, Baldock C. Fibrillin microfibrils and elastic fibre proteins: Functional interactions and extracellular regulation of growth factors. Semin Cell Dev Biol. 2019;89:109–117. Mamm Innate Immun Fungal Infect. 10.1016/j.semcdb.2018.07.016. 10.1016/j.semcdb.2018.07.016 PMC6461133 30016650

[31] Grant AO. Cardiac Ion Channels. Circ Arrhythmia Electrophysiol. 2009;2(2):185–94. 10.1161/CIRCEP.108.789081. 10.1161/CIRCEP.108.789081 19808464

[32] Conesa A, Götz S, García-Gómez JM, Terol J, Talón M, Robles M. Blast2GO: a universal tool for annotation, visualization and analysis in functional genomics research. Bioinformatics. 2005;21(18):3674–6. 10.1093/bioinformatics/bti610. 16081474 10.1093/bioinformatics/bti610

[33] Kreft L, Botzki A, Coppens F, Vandepoele K, Van Bel M. PhyD3: a phylogenetic tree viewer with extended phyloXML support for functional genomics data visualization. Bioinformatics. 2017;33(18):2946–7. 10.1093/bioinformatics/btx324. 28525531 10.1093/bioinformatics/btx324

[34] Eizenga JM, Novak AM, Sibbesen JA, Heumos S, Ghaffaari A, Hickey G, et al. Pangenome graphs. Ann Rev Genomics Hum Genet. 2020;21(1):139–62. 10.1146/annurev-genom-120219-080406. 32453966 10.1146/annurev-genom-120219-080406 PMC8006571

[35] Piñero J, Saüch J, Sanz F, Furlong LI. The DisGeNET cytoscape app: exploring and visualizing disease genomics data. Comput Struct Biotechnol J. 2021;19:2960–7. 10.1016/j.csbj.2021.05.015. 34136095 10.1016/j.csbj.2021.05.015 PMC8163863

[36] Kopanos C, Tsiolkas V, Kouris A, Chapple CE, Albarca Aguilera M, Meyer R, et al. VarSome: the human genomic variant search engine. Bioinformatics. Oxford: 2018;35(11):1978–80. 10.1093/bioinformatics/bty897. 10.1093/bioinformatics/bty897 PMC6546127 30376034

[37] Olivé A. Conceptual modeling of information systems. Springer Science & Business Media; 2017.

[38] Walsh R, Thomson K, Ware J, Funke B, Woodley J, McGuire K, et al. Reassessment Of Mendelian Gene Pathogenicity Using 7,855 Cardiomyopathy Cases And 60,706 Reference Samples. Genet Med. 2016;19. 10.1038/gim.2016.90. 10.1038/gim.2016.90 PMC5116235 27532257

[39] Whiffin N, Walsh R, Govind R, Edwards M, Ahmad MA, Zhang X, et al. CardioClassifier: disease- and gene-specific computational decision support for clinical genome interpretation. Genet Med. 2018;20. 10.1038/gim.2017.258. 10.1038/gim.2017.258 PMC6558251 29369293

[40] Vincent A, Nayar P, Murugesan R, Mary B, P D, Ahmed S. CardioGenBase: A Literature Based Multi-Omics Database for Major Cardiovascular Diseases. PLoS ONE. 2015;10:e0143188. 10.1371/journal.pone.0143188. 10.1371/journal.pone.0143188 PMC4666633 26624015

[41] Collod-Béroud G, Le Bourdelles S, Ades L, Ala-Kokko L, Booms P, Boxer M, et al. Update of the UMD-FBN1 mutation database and creation of an FBN1 polymorphism database. Hum Mutat. 2003;22(3):199–208. 10.1002/humu.10249. 12938084 10.1002/humu.10249

[42] Roberts AM, Ware JS, Herman DS, Schafer S, Baksi J, Bick AG, et al. Integrated allelic, transcriptional, and phenomic dissection of the cardiac effects of titin truncations in health and disease. Sci Transl Med. 2015;7(270):270ra6. 10.1126/scitranslmed.3010134. 25589632 10.1126/scitranslmed.3010134 PMC4560092

[43] O’Mahony C, Jichi F, Pavlou M, Monserrat L, Anastasakis A, Rapezzi C, et al. A novel clinical risk prediction model for sudden cardiac death in hypertrophic cardiomyopathy (HCM Risk-SCD). Eur Heart J. 2013;35(30):2010–20. 10.1093/eurheartj/eht439. 24126876 10.1093/eurheartj/eht439

[44] van der Zwaag PA, Jongbloed JDH, van den Berg MP, van der Smagt JJ, Jongbloed R, Bikker H, et al. A genetic variants database for arrhythmogenic right ventricular dysplasia/cardiomyopathy. Hum Mutat. 2009;30(9):1278–83. 10.1002/humu.21064. 19569224 10.1002/humu.21064

[45] Adler A, Kirchmeier P, Reinhard J, Brauner B, Dunger I, Fobo G, et al. PhenoDis: A comprehensive database for phenotypic characterization of rare cardiac diseases. Orphanet J Rare Dis. 2018;13. 10.1186/s13023-018-0765-y. 10.1186/s13023-018-0765-y PMC5785853 29370821

[46] Nicora G, Limongelli I, Gambelli P, Memmi M, Malovini A, Mazzanti A, et al. CardioVAI: An automatic implementation of ACMG-AMP variant interpretation guidelines in the diagnosis of cardiovascular diseases. Hum Mutat. 2018;39. 10.1002/humu.23665. 10.1002/humu.23665 30298955

[47] LeWinter MM, Granzier HL. Cardiac titin and heart disease. J Cardiovasc Pharmacol. 2014;63(3):207–12. 10.1097/fjc.0000000000000007. 24072177 10.1097/FJC.0000000000000007 PMC4268868

[48] Carrier L, Mearini G, Stathopoulou K, Cuello F. Cardiac myosin-binding protein C (MYBPC3) in cardiac pathophysiology. Gene. 2015;573. 10.1016/j.gene.2015.09.008. 10.1016/j.gene.2015.09.008 PMC6660134 26358504

[49] Morimoto S. Sarcomeric proteins and inherited cardiomyopathies. Cardiovasc Res. 2007;77(4):659–66. 10.1093/cvr/cvm084. 18056765 10.1093/cvr/cvm084

[50] Kobirumaki-Shimozawa F, Oyama K, Serizawa T, Mizuno A, Kagemoto T, Shimozawa T, et al. Sarcomere Imaging by Quantum Dots for the Study of Cardiac Muscle Physiology. J Biomed Biotechnol. 2012;2012:313814. 10.1155/2012/313814. 22570526 10.1155/2012/313814 PMC3335260

[51] Yin Z, Ren J, Guo W. Sarcomeric protein isoform transitions in cardiac muscle: A journey to heart failure. Biochim Biophys Acta (BBA) - Mol Basis of Dis. 2015;1852(1):47–52. 10.1016/j.bbadis.2014.11.003. 10.1016/j.bbadis.2014.11.003 PMC4268308 25446994

[52] Henderson CA, Gomez CG, Novak SM, Mi-Mi L, Gregorio CC. In: Overview of the Muscle Cytoskeleton. Wiley; 2017. pp. 891–944. 10.1002/cphy.c160033. 10.1002/cphy.c160033 PMC5890934 28640448

[53] Knöll R, Buyandelger B, Lab M. The Sarcomeric Z-Disc and Z-Discopathies. J Biomed Biotechnol. 2011;2011:569628. 10.1155/2011/569628. 22028589 10.1155/2011/569628 PMC3199094

[54] Lange S, Pinotsis N, Agarkova I, Ehler E. The M-band: The underestimated part of the sarcomere. Biochim Biophys Acta (BBA) - Mol Cell Res. 2020;1867(3):118440. Cardiomyocyte biology: new pathways of differentiation and regeneration. 10.1016/j.bbamcr.2019.02.003. 10.1016/j.bbamcr.2019.02.003 PMC7023976 30738787

[55] van der Velden J, Stienen GJM. Cardiac Disorders and Pathophysiology of Sarcomeric Proteins. Physiol Rev. 2019;99(1):381–426. PMID: 30379622. 10.1152/physrev.00040.2017. 10.1152/physrev.00040.2017 30379622

[56] Maron BJ, Towbin JA, Thiene G, Antzelevitch C, Corrado D, Arnett D, et al. Contemporary Definitions and Classification of the Cardiomyopathies. Circulation. 2006;113(14):1807–16. 10.1161/CIRCULATIONAHA.106.174287. 16567565 10.1161/CIRCULATIONAHA.106.174287

[57] Krans JL. The sliding filament theory of muscle contraction. Nat Educ. 2010;3(9):66.

[58] Setterberg I, Le C, Frisk M, Perdreau-Dahl H, Li J, Louch W. Corrigendum: The Physiology and Pathophysiology of T-Tubules in the Heart. Front Physiol. 2021;12. 10.3389/fphys.2021.790227. 10.3389/fphys.2021.790227 PMC8576569 34764889

[59] Bartos DC, Grandi E, Ripplinger CM. In: Ion Channels in the Heart. Wiley; 2015. pp. 1423–64. 10.1002/cphy.c140069. 10.1002/cphy.c140069 PMC4516287 26140724

[60] Park DS, Fishman GI. The cardiac conduction system. Circulation. 2011;123(8):904–15. 10.1161/CIRCULATIONAHA.110.942284. 21357845 10.1161/CIRCULATIONAHA.110.942284 PMC3064561

[61] Channelopathies June-Bum K. Korean. J Pediatr. 2014;57(1):1–18. 10.3345/kjp.2014.57.1.1. 10.3345/kjp.2014.57.1.1 PMC3935107 24578711

[62] Campuzano O, Beltrán-Álvarez P, Iglesias A, Scornik F, Pérez G, Brugada R. Genetics and cardiac channelopathies. Genet Med. 2010;12(5):260–7. 10.1097/GIM.0b013e3181d81636. 20386317 10.1097/GIM.0b013e3181d81636

[63] Jana S, Hu M, Shen M, Kassiri Z. Extracellular matrix, regional heterogeneity of the aorta, and aortic aneurysm. Exp Mol Med. 2019;51(12):1–15. 10.1038/s12276-019-0286-3. 31857579 10.1038/s12276-019-0286-3 PMC6923362

[64] Bujak M, Frangogiannis NG. The role of TGF- signaling in myocardial infarction and cardiac remodeling. Cardiovasc Res. 2007;74(2):184–95. 10.1016/j.cardiores.2006.10.002. 10.1016/j.cardiores.2006.10.002 PMC1924687 17109837

